# The proneural transcription factor ASCL1 regulates cell proliferation and primes for differentiation in neuroblastoma

**DOI:** 10.3389/fcell.2022.942579

**Published:** 2022-10-03

**Authors:** Lydia M. Parkinson, Sarah L. Gillen, Laura M. Woods, Lewis Chaytor, Daniel Marcos, Fahad R. Ali, Jason S. Carroll, Anna Philpott

**Affiliations:** ^1^ Department of Oncology, University of Cambridge, Cambridge, United Kingdom; ^2^ Wellcome-MRC Cambridge Stem Cell Institute, Jeffrey Cheah Biomedical Centre, Cambridge Biomedical Campus, Cambridge, United Kingdom; ^3^ College of Medicine, Mohammed Bin Rashid University of Medicine and Health Sciences, Dubai, United Arab Emirates; ^4^ Cancer Research UK Cambridge Institute, University of Cambridge, Cambridge, United Kingdom

**Keywords:** neuroblastoma, ASCL1, differentiation, proliferation, neurogenesis, chromatin accessibility

## Abstract

Neuroblastoma is believed to arise from sympathetic neuroblast precursors that fail to engage the neuronal differentiation programme, but instead become locked in a pro-proliferative developmental state. Achaete-scute homolog 1 (ASCL1) is a proneural master regulator of transcription which modulates both proliferation and differentiation of sympathetic neuroblast precursor cells during development, while its expression has been implicated in the maintenance of an oncogenic programme in MYCN-amplified neuroblastoma. However, the role of ASCL1 expression in neuroblastoma is not clear, especially as its levels vary considerably in different neuroblastoma cell lines. Here, we have investigated the role of ASCL1 in maintaining proliferation and controlling differentiation in both MYCN amplified and Anaplastic Lymphoma Kinase (ALK)-driven neuroblastoma cells. Using CRISPR deletion, we generated neuroblastoma cell lines lacking ASCL1 expression, and these grew more slowly than parental cells, indicating that ASCL1 contributes to rapid proliferation of MYCN amplified and non-amplified neuroblastoma cells. Genome-wide analysis after ASCL1 deletion revealed reduced expression of genes associated with neuronal differentiation, while chromatin accessibility at regulatory regions associated with differentiation genes was also attenuated by ASCL1 knock-out. In neuroblastoma, ASCL1 has been described as part of a core regulatory circuit of developmental regulators whose high expression is maintained by mutual cross-activation of a network of super enhancers and is further augmented by the activity of MYC/MYCN. Surprisingly, ASCL1 deletion had little effect on the transcription of CRC gene transcripts in these neuroblastoma cell lines, but the ability of MYC/MYCN and CRC component proteins, PHOX2B and GATA3, to bind to chromatin was compromised. Taken together, our results demonstrate several roles for endogenous ASCL1 in neuroblastoma cells: maintaining a highly proliferative phenotype, regulating DNA binding of the core regulatory circuit genes to chromatin, while also controlling accessibility and transcription of differentiation targets. Thus, we propose a model where ASCL1, a key developmental regulator of sympathetic neurogenesis, plays a pivotal role in maintaining proliferation while simultaneously priming cells for differentiation in neuroblastoma.

## Introduction

Neuroblastoma is an often devastating paediatric tumour arising from sympathetic noradrenergic neuroblasts of the peripheral nervous system. Tumour cells usually resemble trapped developmental intermediates, which can be stalled at different stages of the developmental trajectory (de Preter et al., 2007; Johnsen et al., 2019; Jansky et al., 2021). Interestingly, there is a subtype of neuroblastoma, the MS type, where young infants present with wide-spread tumours that spontaneously resolve without treatment (D’Angio et al., 1971; Brodeur 2018). This phenomenon is thought to result from re-entry of neuroblasts into a normal developmental trajectory of cell cycle exit and differentiation (Brodeur et al., 1997). However, spontaneous regression does not occur in older children or in high-risk tumours with characteristics such as MYCN amplification or Anaplastic Lymphoma Kinase (ALK) mutation (Ratner et al., 2016; Raitio et al., 2021). These patients are usually treated with high-dose multi-modal chemotherapy, yet still often relapse. A better understanding of why the normal developmental pathway is stalled and corrupted in neuroblastoma is likely to point to new and kinder ways to treat this tumour type that accounts for 15% of all paediatric solid tumour deaths (Maris 2010; Johnsen et al., 2019). To gain this understanding, we must explore how the function of normal developmental regulators of noradrenergic neurogenesis are disrupted in neuroblastoma.

The master regulatory transcription factor Achaete-scute homolog 1 (ASCL1) has a central role in neurogenesis in both the central and peripheral nervous system (C/PNS) (Guillemot et al., 1993; Nieto et al., 2001; Raposo et al., 2015), with a particularly interesting role in balancing proliferation and differentiation. In the CNS, ASCL1 contributes to neural cell “stem-ness” (Castro et al., 2011; Urbán et al., 2016) and also drives neuronal differentiation (Castro et al., 2011; Guillemot and Hassan 2017). ASCL1 is expressed transiently during development, showing upregulation in noradrenergic neuroblasts in the peripheral nervous system, and is downregulated as neurons begin to differentiate (Lo et al., 1991; Wylie et al., 2015). Multiple transcription factors show co-ordinated expression and interact to regulate normal neurogenesis during development (Pattyn et al., 1999; Moriguchi et al., 2006; Morikawa et al., 2007; Rohrer 2011; Lefcort 2020).

ASCL1 can play a paradoxical role in cancers. For instance, ASCL1 has been shown to be both a driver of oncogenesis in glioblastoma (Rheinbay et al., 2013; Vue et al., 2020; Azzarelli et al., 2022) but also to be associated with more favourable prognosis (Park et al., 2017). ASCL1 has a similarly enigmatic role in neuroblastoma. ASCL1 and other developmental regulators of neurogenesis form an aberrant ‘core regulatory circuit’ (CRC), that promotes oncogenicity of neuroblastoma cells (Boeva et al., 2017; van Groningen et al., 2017; Wang et al., 2019), where mutually regulated high levels of expression of the circuit genes is usually reinforced by elevated MYCN (Wang et al., 2019). Higher levels of ASCL1 are associated with poorer neuroblastoma disease outcomes (Wang et al., 2019), and ASCL1 expression in neuroblastoma is negatively correlated with expression of genes involved in terminal neuronal differentiation (Kasim et al., 2016). Elevated and stable ASCL1 levels in neuroblastoma cell lines are consistent with these tumour cells being trapped in a neuroblastic developmental intermediate state (Wylie et al., 2015). In contrast, further enhancing ASCL1 activity by overexpression and by preventing the cell cycle-mediated phosphorylation of ASCL1 protein results in neuroblastoma cells re-entering a developmental trajectory leading to cell cycle exit and differentiation (Ali et al., 2020).

In normal development, ASCL1 is expressed in the bridge cell population, the developmental intermediate group of cells that arises between schwann cell precursors and chromaffin cells, indicating that it acts at a pivotal point in the course of both normal development of this lineage and also in cancer progression (Jansky et al., 2021; Ponzoni et al., 2022). The variability in expression of ASCL1 in different cell lines (Wylie et al., 2015; Alleboina et al., 2021) points to the possibility that different tumours are trapped at various stages of a developmental trajectory in which ASCL1 is usually only transiently expressed, and so could result in ASCL1 playing a pro-proliferative or pro-differentiation function in different cellular contexts. Thus, the role of endogenous ASCL1 in maintaining the oncogenic phenotype and cellular identity of different neuroblastoma cell lines remains unclear.

Here, we wanted to understand the effect of ASCL1 KO on neuroblastoma cell proliferation, differentiation and the impact of ASCL1 KO on the expression and function of the CRC transcription factors. To compare the function of endogenous ASCL1 in different neuroblastoma cell contexts, we have used CRISPR-mediated deletion to compare the effects of ASCL1 deletion in a MYCN-amplified and an ALK-driven neuroblastoma line, investigating cell proliferation and the genome-wide impact on gene expression, chromatin accessibility and on the regulation of the core regulatory circuit genes. Following ASCL1 deletion, cells grow at a slower rate than their parental counterparts. ASCL1 is not essential for transcriptional support of the core regulatory circuit of transcription factors that underpins the oncogenic phenotype. However, ASCL1 does promote binding of the CRC factors Paired-like homeobox 2b (PHOX2B), GATA binding protein 3 (GATA3) and MYC/MYCN to chromatin; a reduction in this binding is likely to lead to the reduced cell growth seen when ASCL1 is deleted. ASCL1 is also required to maintain a more open chromatin configuration around genes associated with neuronal differentiation and promotes their expression. In summary, we find that ASCL1 is required for rapid neuroblastoma cell proliferation and to maintain the strong neuronal identity of neuroblastoma cells.

## Methods

### Cell culture

IMR32 and SH-SY5Y were cultured in DMEM-F12 with l-glutamine (Gibco) supplemented with 10% FBS (Sigma) and 1% Penicillin-Streptomycin (Sigma). Cell lines were validated by submitting a cell pellet for short tandem repeat analysis and the results compared with the data from the Cellosaurus database. Cells were confirmed to be *mycoplasma* negative and were tested at a minimum of every 3 months.

### Generation of ASCL1 knockout lines

ASCL1 KO lines were generated using the CRISPR-Cas9 system. The Cas9-2A-GFP and the U6-BsaI-sgRNA plasmids used for ASCL1 KO were kindly gifted by Prof. Steve Pollard (University of Edinburgh), the design and generation of these plasmids has been previously described (Bressan et al., 2017). The Cas9-2A-GFP and the U6-BsaI-sgRNA plasmid were simultaneously transfected using Lipofectamine 2000 (11668019, Thermo Fisher) and cells were left to recover for 48 h. The transfected cells were sorted into single cells that were expressing GFP 48 h post-transfection to generate clonal lines. These Cas9 positive single cells were subject to colony expansion for 4–6 weeks and each clone was then subject to western blot analysis and sequencing to confirm ASCL1 KO at both the genomic and protein level.

### Proliferation assays

Cell count was completed using the CellCountess II Automated Cell Counter (Thermo Fisher), Trypan Blue cell stain was used to separate the live and dead cell populations. For the growth analysis assays, three biological replicates were measured in duplicate.

### Generation of lentiviral transduced cell lines

Lentiviral transduced lines were generated using the pLVX-CMV-Tet3G system. The generation of the two viruses pLVX-CMV-Tet3G (TET transactivator) and the pLVX-TREG (ASCL1 under control of the TET response element) has been described previously (Ali et al., 2020). ASCL1 KO cells were simultaneously transduced with pLVX-CMV-Tet3G and pLVX-TREG at an MOI of 0.3. To ensure cells contained both virus particles, cells underwent puromycin selection for 1 week followed by G418 selection for 5 days. ASCL1 was induced following doxycycline treatment and protein presence was validated by western blot.

### Western blot/fractionation western blot

Protein was extracted by resuspending cells in RIPA buffer and incubating for 20 min. Debris was removed by centrifuging at 16,000xg for 10 min. Protein lysates were separated on a BisTris gel (Invitrogen), transferred to a nitrocellulose membrane and blocked using 5% milk. Primary antibodies were diluted in 1% milk and incubation took place overnight at 4 °C. Primary antibodies were as follows; ASCL1 (ab211327, abcam), CMYC (ab32072, abcam), GATA3 (D13C9, Cell Signalling Technologies), NMYC (9405, Cell Signalling Technologies), PHOX2B (sc-376997, Santa Cruz), α-Tubulin (66031, ProteinTech), GAPDH (60004, ProteinTech) and Histone H3 (ab1791, abcam). Secondary antibodies were diluted in TBST and incubation took place at room temperature for 1 hour; anti-Mouse (NA931, GE Healthcare) anti-Rabbit (NA934, GE Healthcare)

To analyse protein content in different parts of the cell, the Subcellular Fractionation Kit was used and followed according to manufacturers instructions (78840, Thermo Fisher). Briefly, a fresh cell pellet is resuspended in specific buffers to sequentially extract the cytoplasmic, membrane, soluble nuclear and chromatin bound proteins. Protein isolated was subject to western blot analysis (above).

### RNA extraction and RNA-Seq analysis

Cells were lysed using RLT buffer and RNA extracted following the RNeasy Protocol Part I (Qiagen). RNA-seq samples were poly-A selected with NEB polyA kit and libraries made with NEB Ultra II directional RNA kit. Five biological replicates for each cell line were sequenced. Sequencing was carried out on the NovaSeq (Illumina) and 100bp paired-end reads were generated. Analysis was conducted using an available Snakemake pipeline (Mölder et al., 2021). Reads were trimmed for sequence quality with TrimGalore 0.6.4 using a minimum Phred score cutoff of 20 and aligned to the hg19 genome with STAR 2.6.1a in quant mode (Dobin et al., 2013). DESeq2 version 1.32.0 (Love et al., 2014) was used with batch correction and lfcShrink to identify genes that were differentially expressed (p.adj<0.05) between parental and ASCL1 KO conditions. This was conducted separately for each KO line and the overlap of differential genes in both KOs compared to parental used in downstream analysis. Gene set enrichment analysis was conducted using the clusterProfiler R package (Yu et al., 2012). RNA-Seq data and methods can be found at https://www.ncbi.nlm.nih.gov/geo/ and have been assigned the identifier GSE202634.

### ATAC-seq

For each sample, FACS was used to count 50,000 live cells. The Omni-ATAC protocol was followed precisely (Corces et al., 2017). For each cell line, four biological replicates were prepared and sequenced. Samples were sequenced on the NovaSeq and 50bp paired-end reads were generated. Analysis was conducted using an adapted version of an available Snakemake pipeline (Mölder et al., 2021). Reads were trimmed for sequence quality with TrimGalore 0.6.4 using a minimum Phred score cutoff of 20, and mapped to the hg19 genome with Bowtie2 2.4.1 (Langmead and Salzberg 2012). Peaks were called using Macs2 2.2.7.1 in narrowPeak mode (Zhang et al., 2008). DiffBind version 2.14.0 (Stark and Brown 2011) was used with default parameters and summits = 200 to identify differentially accessible regions between parental and ASCL1 KO lines. These differentially accessible regions were assigned to a gene based on the proximity to a TSS. These gene lists were then used in GO analysis to identify biological processes and cellular components associated with the regions. ATAC-seq track plots were generated using IGV (Robinson et al., 2011). ATAC-Seq data and methods can be found at https://www.ncbi.nlm.nih.gov/geo/and have been assigned the identifier GSE202629.

Heatmaps of average signal intensity across the 4 replicates (normalised by RPGC–reads per genomic coverage) surrounding ATAC-seq peak summits were generated using deepTools (Ramírez et al., 2014). The heatmaps in Supplementary Figure S4 are ordered by the adjusted *p*-value (FDR) for the significance of the change in accessibility at the genomic region and are all centred around the summit of the regions with differential accessibility from the ATAC-seq. For comparison with SH-SY5Y ASCL1 ChIP-seq, clone WT51 uninduced and induced ASCL1 overexpression data was used from Ali et al., 2020 - GSE153823 (RPKM normalised). For plotHeatmap zMax was set to the 95th percentile of intensity values to prevent outliers from causing extreme skew to the heatmap scaling.

PHOX2B and GATA3 ChIP-seq datasets in parental BE2C and Kelly neuroblastoma cell lines were obtained from Durbin et al., 2018 (GSE94822). These ChIP-seq peaks and the ATAC-seq regions were associated with a gene by assigning the closest TSS using the ChIPseeker R package (Yu et al., 2015).

### Motif analysis

Motif analysis was conducted using HOMER findMotifsGenome.pl to find *de novo* motifs (Heinz et al., 2010). Regions were separated by either increased or decreased accessibility in SH-SY5Y ASCL1 knockout cells and then further separated by the presence or absence of an endogenous ASCL1 binding site using SH-SY5Y ChIP-seq data from Ali et al., 2020 (GSE153823). The similar binding motifs represent proteins with known binding motifs that have been curated for high similarity to the discovered *de novo* motifs.

### Data availability

All the ATAC-seq and RNA-seq data used for this publication have been deposited on GEO https://www.ncbi.nlm.nih.gov/geo/. The RNA-seq data has been assigned the identifier GSE202634 and the ATAC-seq data has been assigned the identifier GSE202629.

## Results

### ASCL1 deletion results in slower growth of neuroblastoma cells

**FIGURE 1:**
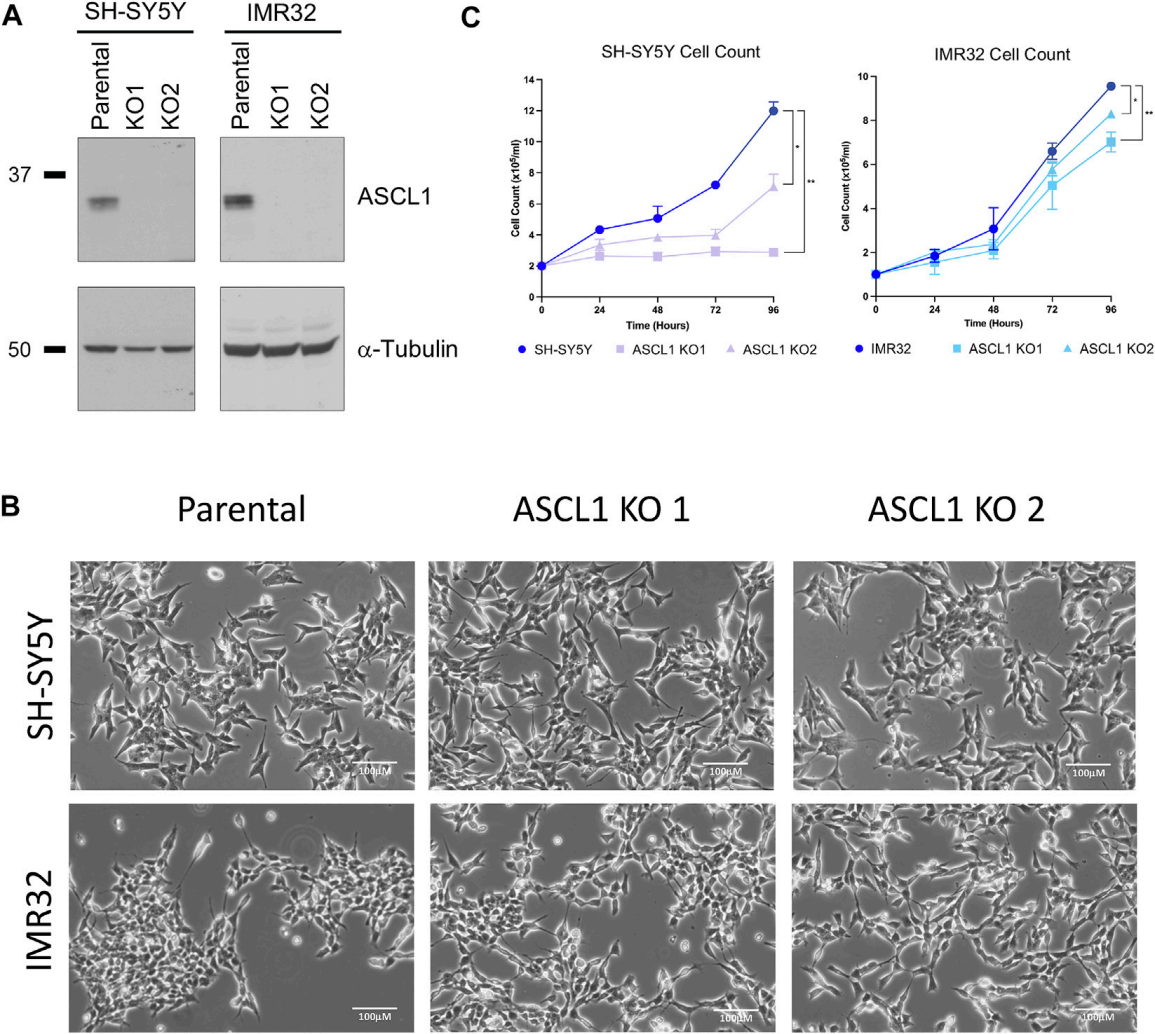
ASCL1 was successfully knocked out of two neuroblastoma lines. **(A)** CRISPR-Cas9 was used to delete ASCL1 from SH-SY5Y and IMR32 cell lines. Western blotting to show the ASCL1 expression levels in parental lines and in lines following ASCL1 KO, α-tubulin was used as the loading control (SH-SY5Y left and IMR32 right). **(B)** Phase contrast microscopy images to show the morphology of cells following ASCL1 KO. **(C)** Cell count data of parental and ASCL1 KO cells over a 96-h period. n = 3, graphs show mean value and ± SEM (unpaired two-tailed *t*-test * = *p* < 0.05, ** = *p* < 0.005; SH-SY5Y left and IMR32 right).

To probe the role of endogenous ASCL1 in maintaining the proliferating adrenergic phenotype of neuroblastoma cells, we investigated the effect of ASCL1 deletion in two neuroblastoma lines; the MYCN-amplified IMR32 cell line and the non-MYCN amplified SH-SY5Y cell line, which both express similar levels of ASCL1 (Supplementary Figure S1A). Using CRISPR/Cas9, we permanently deleted the ASCL1 gene from SH-SY5Y and IMR32 cells targeting deletion from upstream of the bHLH DNA binding region to ensure mutations would result in loss of protein activity (Supplementary Figure S1B). Following CRISPR knock-out (KO), cells were sorted, clonally expanded and screened via sequencing and western blot to demonstrate effective gene targeting (Supplementary Figure S1C). Two ASCL1 KO clones for each cell line were chosen for further experimentation (Figure 1A).

ASCL1 is known to support an adrenergic (ADRN) identity, a subtype of neuroblastoma associated with more neuronal features that is usually morphologically distinct from an alternative mesenchymal (MES) subtype (van Groningen et al., 2017). ASCL1 does not appear to be required to maintain ADRN neuroblastoma cell morphology; in both cell lines, ASCL1 KO cells look similar to their parental ASCL1-expressing counterparts (Figure 1B), albeit IMR32 KO cells tend to clump together less readily than the parental line. To determine the extent to which ASCL1 expression contributes to the rapidly proliferating phenotype of neuroblastoma cells, we compared the cell number of parental and ASCL1 KO cell lines, expanding over time. In both cell lines, there was a small but significant reduction in the number of cells over time where ASCL1 had been deleted compared to parental control lines (Figure 1C). We also deleted ASCL1 from an additional neuroblastoma cell line, SK-N-BE(2)C (Supplementary Figure S1D) and saw the same slower growth phenotype (Supplementary Figure S1E). In all ASCL1 KO lines, cell growth is slower although we have not assayed tumorigenicity directly.

### ASCL1 deletion results in the downregulation of genes associated with differentiation

ASCL1 is known to be a pioneer factor able to open closed chromatin and a master regulator of transcription driving neuronal differentiation in embryonic development (Wapinski et al., 2013; Raposo et al., 2015). It is also an important member of the so-called “core regulatory circuit” of transcription factors that determines the ADRN identity of aggressive neuroblastoma tumours (Wang et al., 2019). To probe the genome-wide transcriptional consequences of deletion of endogenous ASCL1, we next compared the transcriptomes of exponentially growing parental and ASCL1 KO cells by RNA-seq.

**FIGURE 2:**
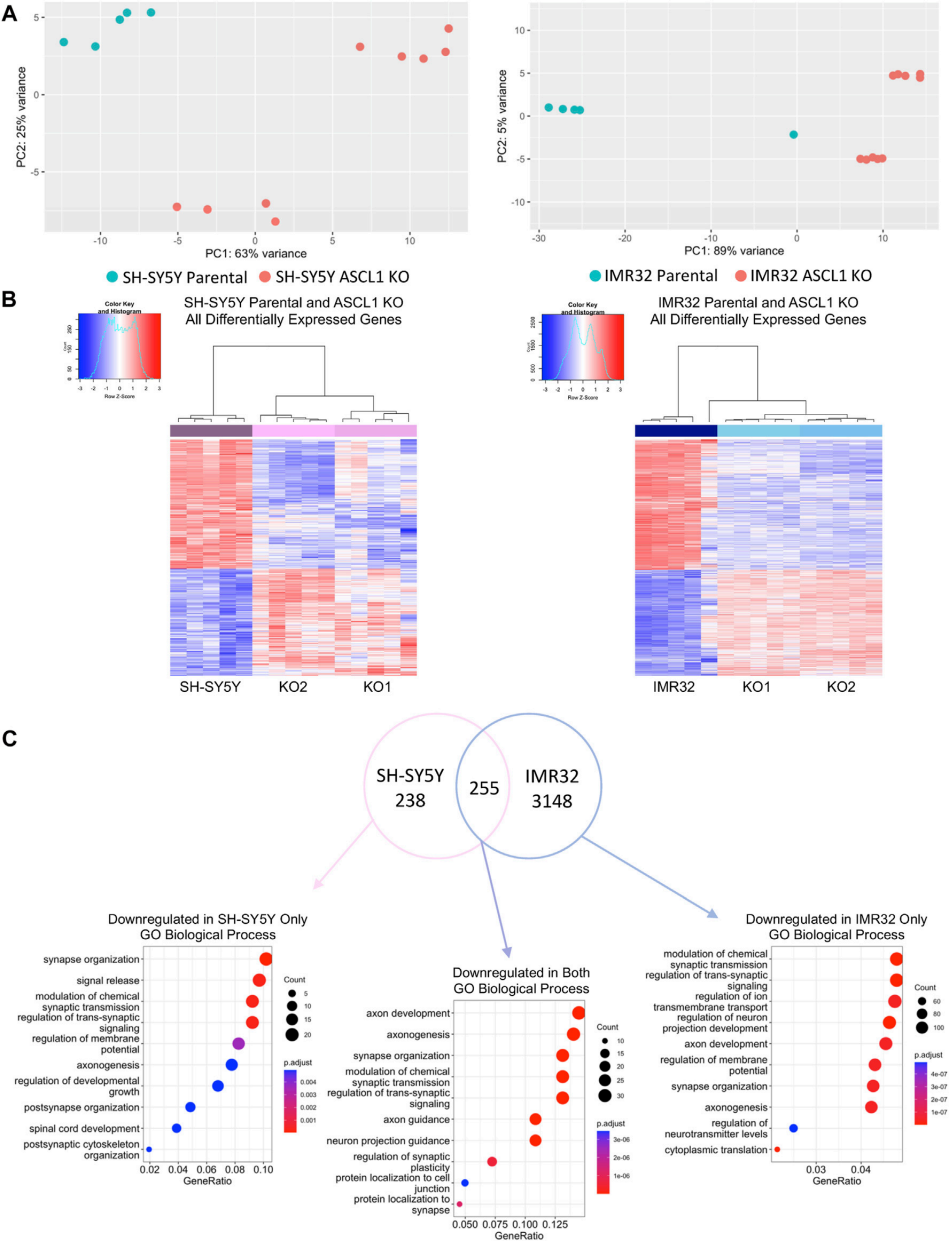
ASCL1 knockout results in downregulation of differentiation target genes. **(A)** PCA plot to show the differences between SH-SY5Y parental and ASCL1 KO lines (left) and IMR32 parental and ASCL1 KO lines (right). **(B)** Heatmaps to show differentially expressed genes following ASCL1 KO in SH-SY5Y and IMR32 cell lines. **(C)** Venn diagram to show the genes downregulated in SH-SY5Y ASCL1 KO lines, IMR32 ASCL1 KO lines and the genes downregulated in both (FDR<0.05, LogFC < -0.25). Gene Ontology analysis was completed on each gene set. The top 10 GO terms associated with biological process are shown. RNA-Seq was completed on 5 biological replicates.

Principal Component Analysis (PCA) comparing each parental cell line with the corresponding ASCL1 KO cells showed significant transcriptional differences resulting from gene deletion (Figure 2A). In IMR32 cells, 3403 genes were significantly downregulated following ASCL1 knockout, while 2865 genes were upregulated (Figures 2B,C; Supplementary Figure S2B). In ASCL1 KO SH-SY5Y cells, 493 genes were downregulated and 409 upregulated compared to parental controls (Figures 2B,C; Supplementary Figure S2B). ASCL1 is generally accepted to be a transcriptional activator, and this has been confirmed in neuroblastoma cells by our previous analysis of changes in gene expression in response to ASCL1 overexpression (Woods et al., 2022). Only 94 genes were upregulated across duplicates of both cell lines following ASCL1 KO, and these changes are likely to be an indirect result of gene knock-out (Supplementary Figure S2B). We compared the list of downregulated genes in each cell line to previous ChIP-seq data identifying ASCL1 binding sites after ASCL1 overexpression in SH-SY5Y cells (Ali et al., 2020; Woods et al., 2022) and found 79% of downregulated genes in SH-SY5Y KO lines and 72% downregulated genes in IMR32 KO lines were associated with one or more ASCL1-binding site (Supplementary Figure S2C).

We next explored the function of genes that were downregulated after ASCL1 KO, as effects on these genes were likely to be direct effects of ASCL1 deletion. To explore this, Gene Set Enrichment Analysis (GSEA) was conducted on genes downregulated in ASCL1 KO cells compared to parental cells in both cell lines, as well as genes downregulated specifically in 1 cell line or the other (Figure 2C). This analysis showed genes downregulated after ASCL1 KO were associated with neuronal differentiation with many biological processes, such as axonogenesis, synapse organisation and synaptic signalling, shared by both cell lines (Figure 2C). Consistent with a role for ASCL1 in supporting a neural identity in neuroblastoma cells, genes downregulated on ASCL1 KO that were also associated with an ASCL1 binding peak in our previous ASCL1 over-expression analysis (Ali et al., 2020; Woods et al., 2022) were also involved in neurogenesis (Supplementary Figure S2D). We saw that GO terms associated with genes upregulated after ASCL1 KO included categories involved in metabolism and organelle control (Supplementary Figure S2B). Because ASCL1 is a transcriptional activator (Raposo et al., 2015; Woods et al., 2022) these likely represent indirect effects of ASCL1 KO.

### ASCL1 loss results in less accessible chromatin around differentiation genes

**FIGURE 3:**
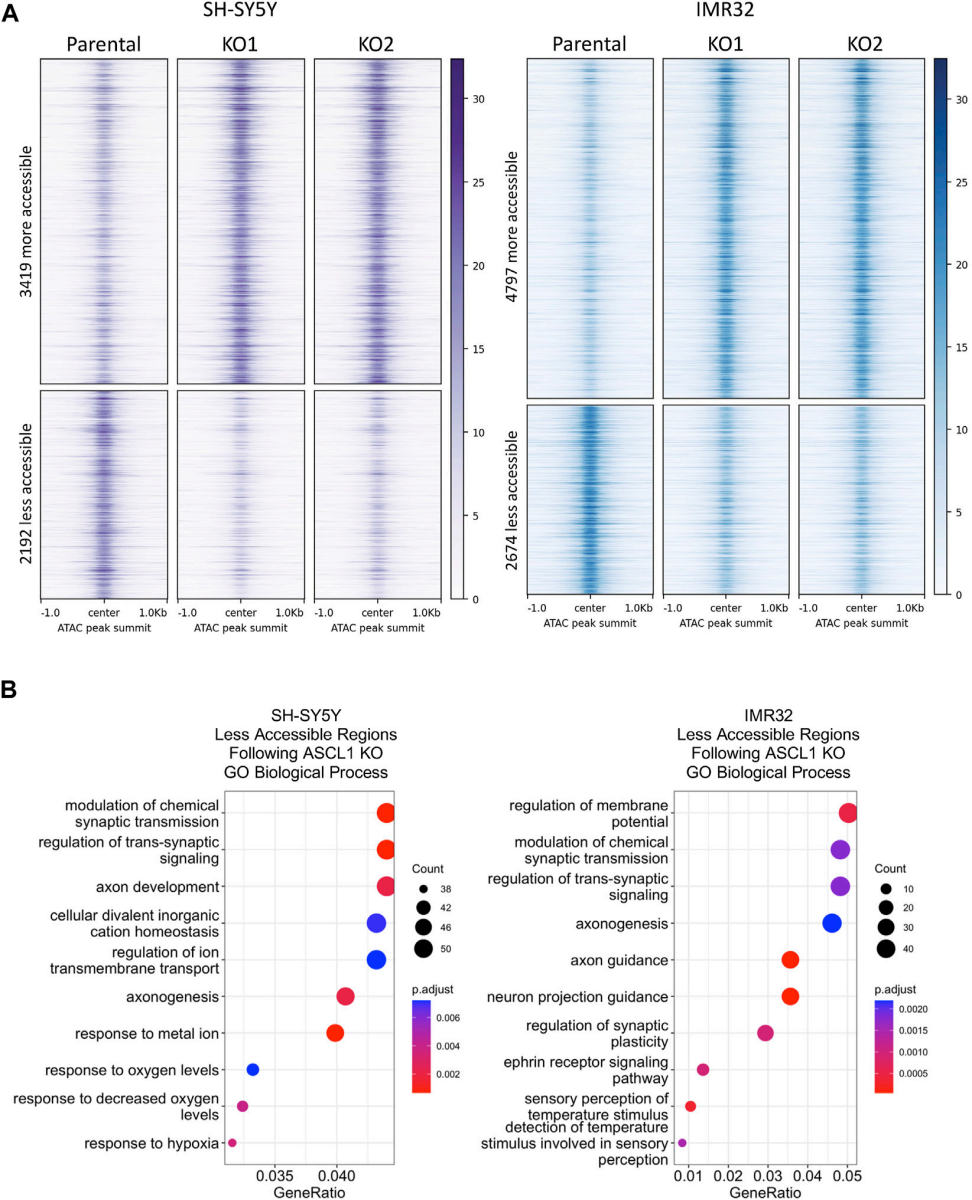
Differentiation targets are less accessible in ASCL1 KO lines. **(A)** Signal intensity −/+1kb around peak summits of all regions with significantly altered accessibility in ASCL1 KO cell lines, for SH-SY5Y cells (purple) and IMR32 cells (blue). ATAC-seq data is RPGC normalised and the average of four replicates. **(B)** Genes were identified that were within 50 kb of an ATAC region with lower accessibility following ASCL1 KO. Gene Ontology analysis was performed on these gene sets, SH-SY5Y (left) and IMR32 (right). The top 10 GO terms are shown. ATAC-Seq completed on 4 biological replicates.

ASCL1 can act as a pioneer factor and its binding is needed for subsequent recruitment of BRN2 and MYT1L in reprogramming of fibroblasts to neurons (Wapinski et al., 2017), indicating that ASCL1 can modulate chromatin to regulate binding of non-pioneer factors to enhance neurogenesis. As described above, we find that deletion of ASCL1 results in downregulation of genes associated with more mature neuronal function in neuroblastoma cells (Figure 2). We utilised Assay for Transposase Accessible Chromatin (ATAC)-Seq to determine if the downregulation of neuronal genes was a consequence of ASCL1 KO resulting in reduced accessibility around these genes. We generated ATAC-seq data comparing parental and ASCL1 KO lines and used DiffBind (Stark and Brown 2011) to identify differentially accessible regions of chromatin. ASCL1 KO lines show multiple regions with altered chromatin accessibility compared to the parental lines (Figure 3A), demonstrating a significant effect of ASCL1 deletion on chromatin configuration genome-wide. We saw that many of the regions are distal from transcriptional start sites (Supplementary Figure S3A).

**FIGURE 4:**
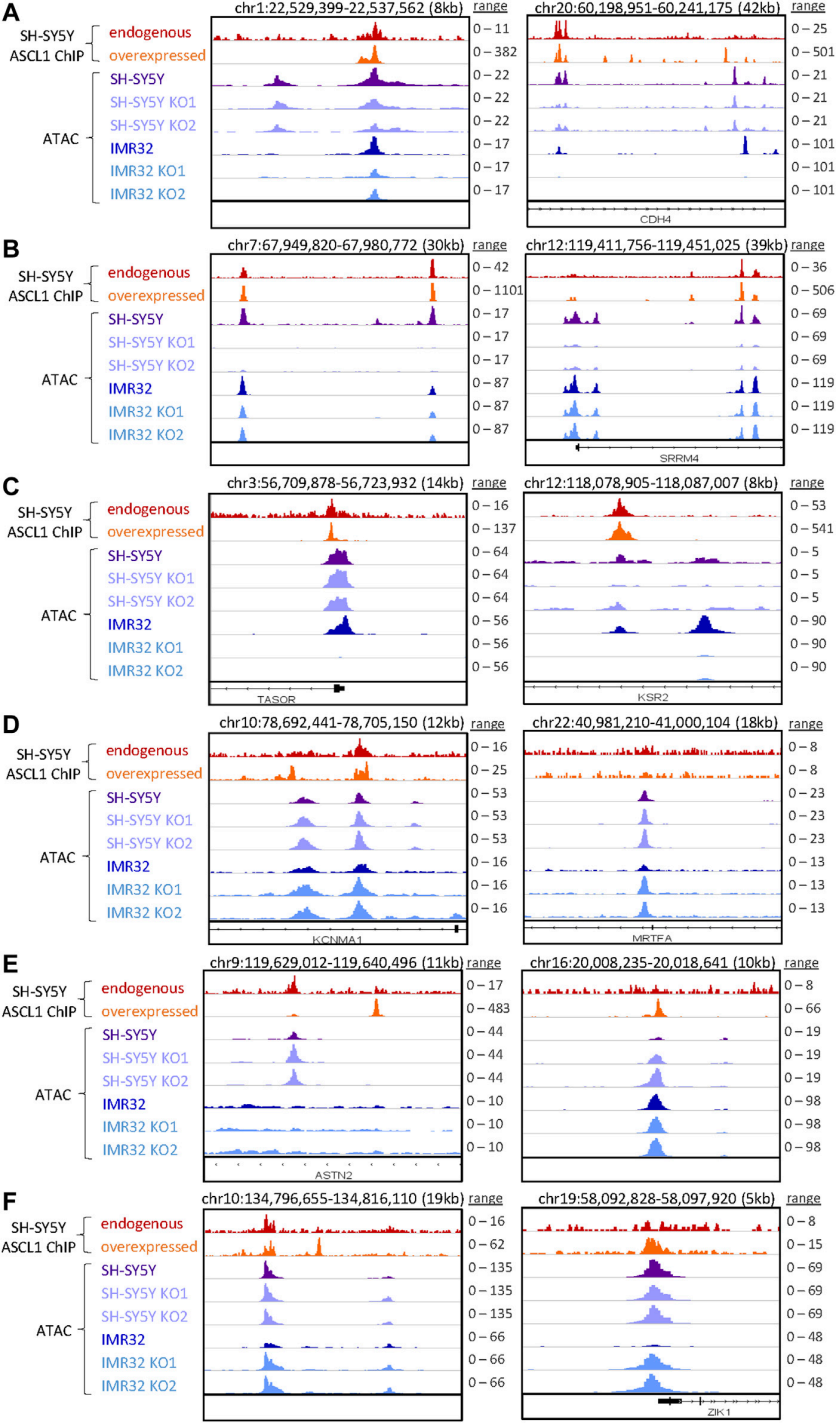
Specific regions of altered accessibility after ASCL1 knockout. Genome browser tracks show examples of specific regions with altered accessibility (accessibility is shown in purple for SH-SY5Y and blue for IMR32 cells, RPGC normalised) and the corresponding ASCL1 binding (red is endogenous ASCL1 binding, and orange shows the binding of overexpressed WT ASCL1, RPKM normalised data from Ali et al., 2020). In each plot the scales are consistent for the ATAC-seq data within each cell line, while for the ASCL1 ChIP-seq data the scales are set to the max peak height for endogenous and overexpressed ASCL1 independently. Example regions shown are: **(A)** decreased accessibility in SH-SY5Y and IMR32 **(B)** decreased accessibility in SH-SY5Y only **(C)** decreased accessibility in IMR32 only **(D)** increased accessibility in SH-SY5Y and IMR32 **(E)** increased accessibility in SH-SY5Y only **(F)** increased accessibility in IMR32 only.

ASCL1 has a well-documented role as a pioneer factor opening closed chromatin during neurogenesis and reprogramming (Raposo et al., 2015; Wapinski et al., 2017), so we first focussed on areas of the genome where accessibility is reduced after ASCL1 knock-out. We took the list of regions showing reduced accessibility in ASCL1 KO compared to parental cells and assigned regulated genes as those within 50 kb of these areas. We then performed GO analysis to identify biological processes that are likely to be affected by ASCL1 KO-mediated changes in chromatin accessibility. Regions with reduced accessibility following ASCL1 deletion were associated with genes involved in neuronal differentiation and development such as synaptic signalling and axonogenesis (Figure 3B). We next investigated regions with reduced accessibility that were common in all 4 ASCL1 KO lines and regions that were cell line-specific (Supplementary Figure S3B), examples of which are shown in Figures 4A–C. GO analysis did not identify any statistically significant terms from the sites that were shared between all 4 KO lines. When probing sites that were cell line-specific, GO analysis showed that the less accessible regions following ASCL1 KO in SH-SY5Y cells are associated with axon development and the synapse (Supplementary Figure S3B). Similarly, following ASCL1 KO in IMR32 cells, less accessible regions are associated with the axon, synapse and neuron projection (Supplementary Figure S3B). This is consistent with ASCL1’s known role in developmental neurogenesis and mirrors our RNA-Seq results that show a reduction in expression of genes associated with more mature neuronal functions after ASCL1 deletion. Thus, ASCL1 functions to maintain chromatin accessibility of genes associated with neuronal identity and function in neuroblastoma cell lines.

We also compared regions that were more accessible following ASCL1 KO in all 4 cell lines as well as those regions that were cell line specific (Supplementary Figure S3C), specific examples of which are shown in Figures 4D–F. Regions that were more accessible following ASCL1 in SH-SY5Y cells included those associated with actomyosin structure organisation and stress fibre assembly, while more accessible regions in IMR32 KO lines included those associated with muscle system process and mesenchymal cell differentiation.

To investigate further the direct and indirect involvement of ASCL1 in regulating the chromatin accessibility changes, we used available ChIP-seq data from endogenous (uninduced) and overexpressed ASCL1 (Ali et al., 2020) to look at the localisation of ASCL1 binding sites in relation to the regions are differentially accessible following ASCL1 KO. This shows a clear enrichment for ASCL1 binding directly at the summits of the regions that have altered accessibility in ASCL1 KO cells (Supplementary Figure S4A), suggesting the pioneer factor ASCL1 directly plays a role in regulating chromatin accessibility of these regions.

ASCL1 has a well-documented role as a pioneer factor but its role as a transcriptional repressor has not been widely described. To identify possible regulatory binding partners of ASCL1 or indirect regulators motif analysis was conducted using HOMER on the differentially accessible regions separated by the presence of absence of an endogenous ASCL1 binding site at the region (Supplementary Figure S4B-E, top 5 *de novo* motifs shown). Notably, this identified motifs with high similarity to the known binding motifs of several neuroblastoma core regulatory circuit transcription factor motifs including HAND2, GATA3 and PHOX2B. These analyses are consistent with ASCL1 working in part by controlling the activity of other members of the transcriptional core regulatory circuit in neuroblastoma (Durbin et al., 2018).

### ASCL1 deletion inhibits chromatin binding of key core regulatory circuit transcription factors

**FIGURE 5:**
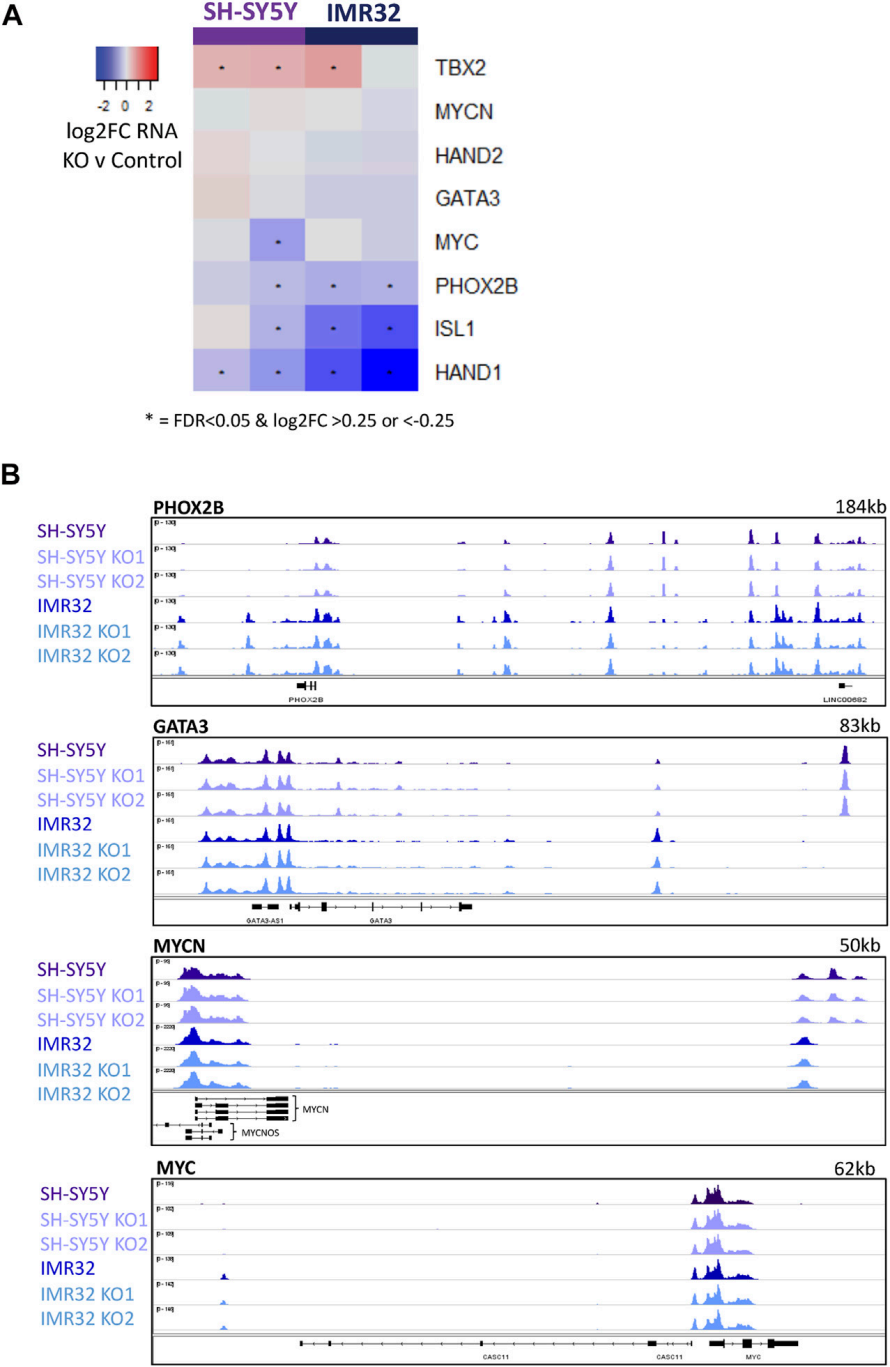
ASCL1 KO does not have an effect on CRC transcription. **(A)** Heatmap highlighting the change in RNA expression of key CRC genes in ASCL1 KO lines compared to parental, * indicates significant genes with a p. adj<0.05 and a log2FC > 0.25/<-0.25. **(B)** ATAC-Seq tracks showing regions around the key TFs PHOX2B, GATA3, MYCN and MYC. ATAC-Seq was completed on 4 biological replicates.

The adrenergic identity of the ADRN subtype of neuroblastoma cells is maintained by a transcriptional core regulatory circuit (CRC) of predominantly developmental regulators of neurogenesis including PHOX2B, GATA3 and ASCL1, whose expression is further maintained by high levels of MYC or MYCN (Boeva et al., 2017; van Groningen et al., 2017; Wang et al., 2019). Previous studies describe this network of mutually reinforcing transcription factors as acting at associated super-enhancer regions to ensure maintenance of high-level CRC component expression, which is thought to reinforce the rapidly proliferating neuroblastic and oncogenic phenotype (Boeva et al., 2017; van Groningen et al., 2017). Wang *et al* saw that downregulation of ASCL1 using siRNA from its usual very high level in Kelly neuroblastoma cells to a more modest level, resulted in a decrease in the expression of PHOX2B and GATA3 (Wang et al., 2019), supporting a role for ASCL1 in maintenance of the CRC. However, this was not observed by others following ASCL1 knock-down in the same cell line (Alleboina et al., 2021). To explore this further, we interrogated the effect of ASCL1 deletion on expression of CRC components as defined previously (Boeva et al., 2017; van Groningen et al., 2017; Wang et al., 2019). In contrast to the previously described siRNA knock-down study (Wang et al., 2019), we saw very limited and mostly insignificant changes in CRC gene expression, with some variation between cell lines (Figure 5A).

As ASCL1 KO cells grow more slowly than parental lines and growth is reported to be supported by this CRC network, Wang et al. had suggested that knock-down of ASCL1 in Kelly cells led to suppression of GATA3 and PHOX2B transcription, which might have explained this slower growth (Wang et al., 2019). However, after ASCL1 KO in IMR32 and SH-SY5Y cells we saw only small and generally insignificant effects on transcription of CRC genes (Figure 5A). Although there are some differences in the transcription of selected CRC transcription factors, the changes are less pronounced than previous studies have reported (Wang et al., 2019). We then interrogated the accessibility of chromatin around these key ADRN CRC transcription factors in cells with and without ASCL1. Consistent with our observation that ASCL1 KO has little effect on PHOX2B, GATA3, and MYC/MYCN gene transcript levels (Figure 5A), ATAC-seq analysis showed that ASCL1 deletion had little effect on chromatin accessibility at the CRC genes (Figure 5B, Supplementary Figure S5). This finding is consistent with the association of CRC genes with open highly transcribed chromatin. ASCL1 has previously been described to be a component of the CRC (Wang et al., 2019) but there is an expected level of redundancy, as is indicated by the modest effect of ASCL1 deletion on expression of most of these genes.

**FIGURE 6:**
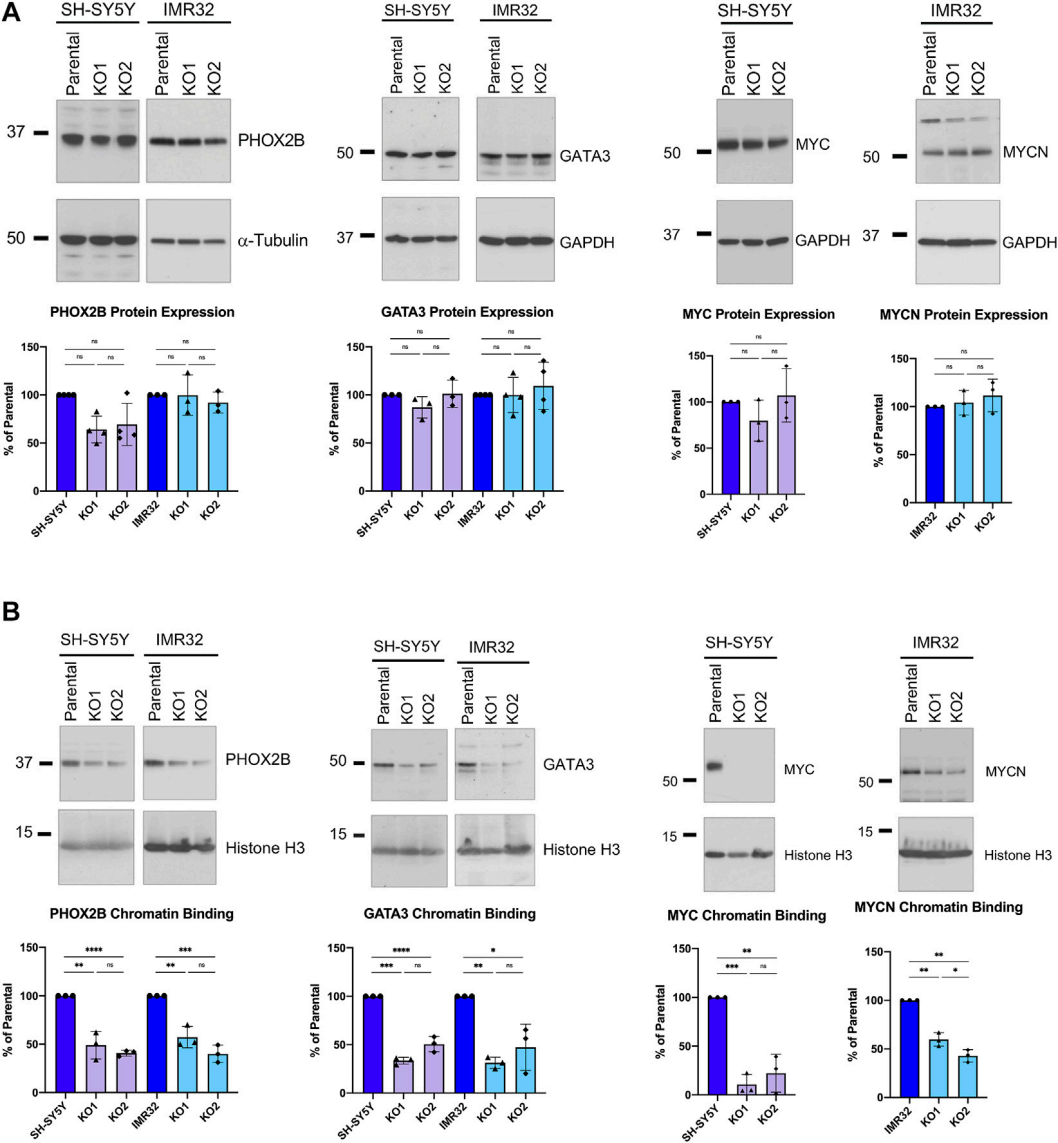
ASCL1 KO results in less CRC transcription factor chromatin binding. **(A)** Western blots showing the overall PHOX2B protein levels (left) and GATA3 levels (centre) and MYC/MYCN levels (right) in parental and ASCL1 KO cells. α-tubulin was used as the loading control for PHOX2B and GAPDH was used as the loading control for GATA3 and MYC/MYCN. n = 3, graphs show all data points and the mean value, two tailed unpaired *t*-test (ns = p = > 0.05). **(B)** Western blot to show PHOX2B (left), GATA3 (middle) and MYC/MYCN (right) levels within the chromatin fraction in parental and ASCL1 KO cells. Histone H3 was used as the control for the chromatin fraction. n = 3, graphs show all data points and the mean value, two tailed unpaired *t*-test (ns = p = > 0.05, * = *p* < 0.05, ** = *p* < 0.005, *** = *p* < 0.0005, ****p = < 0.0001).

Given the well-documented role of these CRC genes in maintaining the rapidly proliferating phenotype of neuroblastoma cells (Boeva et al., 2017; Durbin et al., 2018) and the reduced proliferation we saw after ASCL1 deletion, we then looked to see if ASCL1 KO was affecting the level and/or activity of PHOX2B and GATA3 proteins using western blotting. We did not detect a significant decrease in overall PHOX2B or GATA3 protein levels in ASCL1 KO lines compared to parental cells (Figure 6A). As well as developmental regulators of neurogenesis, the MYC or MYCN oncogenes play a key role in maintaining the high level of cell growth in neuroblastoma tumours; the proliferation of SH-SY5Y cells is supported by MYC and the proliferation of IMR32 is driven by MYCN (Vandesompele et al., 2003; Zimmerman et al., 2018). We investigated whether ASCL1 KO resulted in a reduction in protein expression of MYC/MYCN. As we had seen for PHOX2B and GATA3, the overall protein levels of MYC/MYCN remain very similar following ASCL1 KO (Figure 6A).

ASCL1 acts as a pioneer factor and is required to bind to chromatin initially, allowing recruitment of other neurogenic factors when undergoing transcription factor-mediated reprogramming of fibroblasts to neurons (Wapinski et al., 2013). We next investigated whether deletion of ASCL1 affects binding of PHOX2B, GATA3, transcription factors that are expressed transiently in development to affect the normal developmental cascade of gene expression seen during neurogenesis (Rohrer 2011; van Groningen et al., 2017; Durbin et al., 2018) and/or MYC/MYCN to chromatin. Using western blotting to detect chromatin bound protein in parental and ASCL1 KO lines, deletion of ASCL1 led to a clear reduction in chromatin binding of PHOX2B, GATA3 and MYC/MYCN in both cell lines (Figure 6B). Also, motif analysis of the differentially accessible regions in ASCL1 KO lines (Supplementary Figure S4E) identified possible PHOX2B and GATA3 binding sites, suggesting that endogenous ASCL1 is impacting the chromatin binding of these proteins.

### Reintroduction of ASCL1 to ASCL1 KO cells restores CRC factor binding to chromatin

**FIGURE 7:**
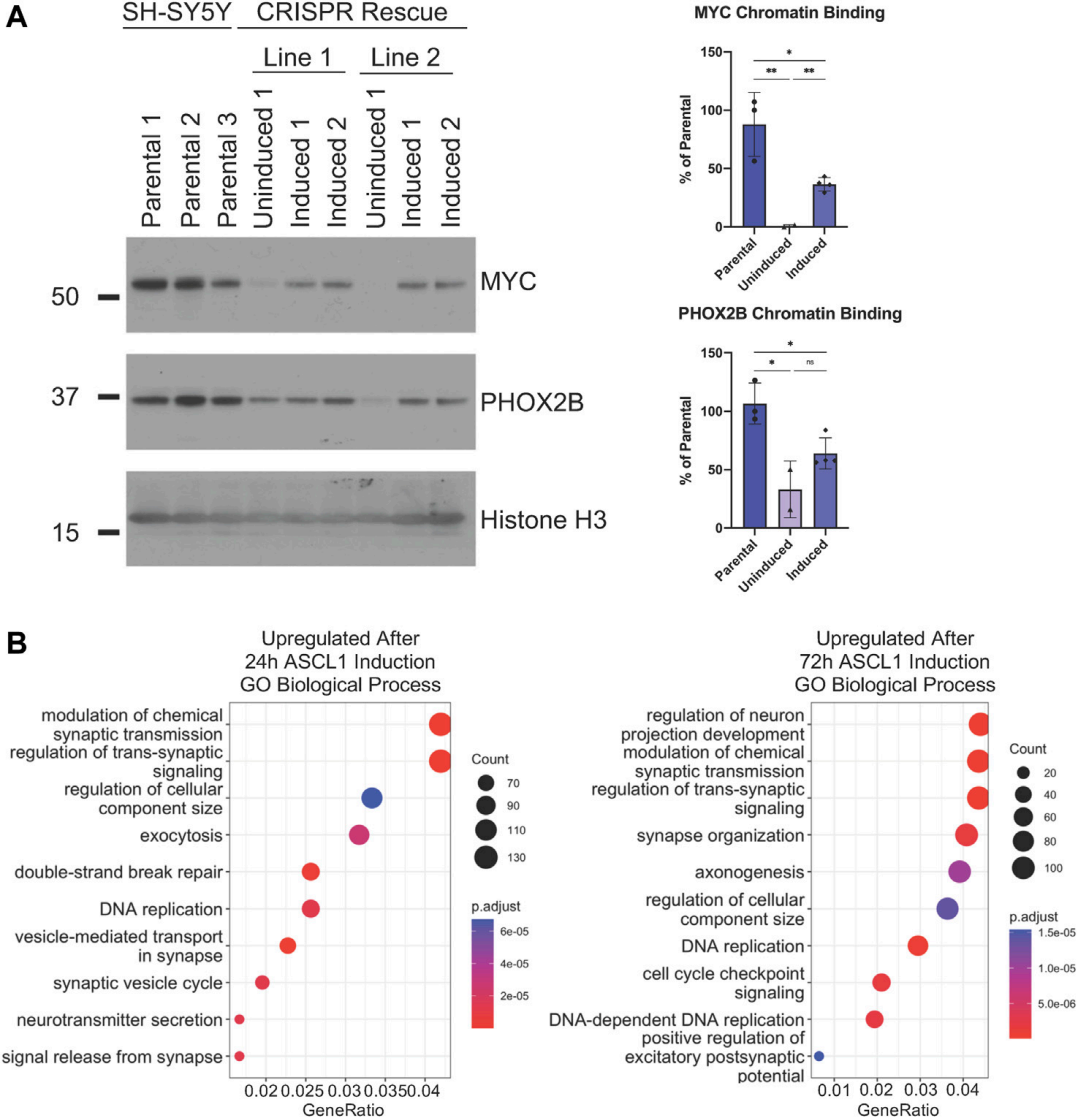
ASCL1 is needed for CRC transcription factor chromatin binding. **(A)** Western blot to show MYC and PHOX2B levels within the chromatin fraction in parental, uninduced ASCL1 rescue cells and following ASCL1 induction into rescue lines (rescue = ASCL1 KO lines with inducible ASCL1. ASCL1 induced using 5 ng Doxycycline). Histone H3 was used as the control for the chromatin fraction. n = 3, graphs show all data points and the mean value, two tailed unpaired *t*-test (ns = p = > 0.05, * = *p* < 0.05, ** = *p* < 0.005, *** = *p* < 0.0005, ****p = < 0.0001). **(B)** RNAseq was completed and GO analysis was performed on the genes identified as differentially expressed when comparing ASCL1 KO and ASCL1 induction rescue cells. The top 10 upregulated (more transcribed following ASCL1 reintroduction) GO terms are shown.

In order to confirm that reduced binding of MYC and PHOX2B to chromatin we had observed following ASCL1 knockout could be directly attributed to ASCL1 deletion, we prepared clonal ASCL1 KO cell lines where a doxycycline-inducible allele of ASCL1 had been reintroduced to enable us to re-express ASCL1 in KO cells at a level resembling endogenous expression (Supplementary Figure S6A). Re-expression of ASCL1 resulted in a significant enhancement of MYC binding to chromatin compared to the uninduced ASCL1 KO lines confirming regulation by ASCL1 (Figure 7A). As MYC is a master driver of the oncogenic phenotype in neuroblastoma and acts as an amplifier of the expression of multiple downstream targets that promote proliferation, this is likely to underpin many of the phenotypic changes observed. There is also an increase in PHOX2B binding following ASCL1 re-expression, albeit at a level that does not reach statistical significance (Figure 7A).

We then investigated whether restoration of ASCL1 expression could rescue expression of neuronal differentiation-associated genes that are downregulated in ASCL1 KO cells (Figure 2). The transcriptome of ASCL1 KO cells was compared with rescue cell lines where ASCL1 had been re-expressed in ASCL1 KO cells for 24 or 72 h. We focussed on genes which were upregulated following ASCL1 induction as their upregulation was likely to be the direct result of ASCL1 expression, undertaking GSEA analysis to identify biological processes associated with this gene set. Following 24 h of ASCL1 reintroduction, upregulated genes were associated particularly with the synapse and neurotransmitter release, while 72 h of ASCL1 induction resulted in upregulated genes associated with synaptic signalling, axonogenesis and neuron projection (Figure 7B). When focussing on GO analysis of cellular components, terms associated with the synapse and axon were identified (Supplementary Figure S6B). These data confirm the requirement for ASCL1 in maintaining a more mature neuronal identity in neuroblastoma cells.

## Discussion

ASCL1 has been described as having both a growth promoting (Wang et al., 2019; Vue et al., 2020) and growth suppressing role in human cancers (Park et al., 2017; Azzarelli et al., 2022). This mirrors its dual role in stem/progenitor cell maintenance as well as neuronal differentiation in the central nervous system (Castro et al., 2011; Raposo et al., 2015). Our previous studies have shown that overexpression of ASCL1 in the SH-SY5Y neuroblastoma cell line is sufficient to promote expression of differentiation-associated genes (Ali et al., 2020; Woods et al., 2022). Additional activation by preventing the CDK-dependent phosphorylation of ASCL1 further engages an extensive programme of cell cycle exit and terminal differentiation (Ali et al., 2014; Ali et al., 2020), indicating that the level of ASCL1 is critical to determining the balance between proliferation and differentiation in neuroblastoma. Here, we have shown in MYCN- and ALK- driven cell lines that endogenous ASCL1 plays a dual function in neuroblastoma, contributing to a rapidly proliferating progenitor identity while maintaining chromatin accessibility at loci associated with differentiation, and so acting in a key role to control the balance between proliferation and differentiation.

In normal development, ASCL1 is transiently expressed with levels rising during noradrenergic neuronal specification, then falling rapidly on neuronal differentiation (Lo et al., 1991; Wylie et al., 2015). Indeed, recent single cell RNA-seq analysis characterising this developmental process revealed that ASCL1 is most highly expressed in the so-called bridge cell population which is found between Schwann cell precursors and chromaffin type cells along the proposed developmental trajectory (Jansky et al., 2021; Ponzoni et al., 2022). It has been suggested that neuroblastoma, a notoriously heterogeneous disease, may arise from progenitors stalled at different points along this developmental pathway, which is consistent with the very heterogeneous expression of ASCL1 seen in different cell lines (Wylie et al., 2015; Alleboina et al., 2021). Thus, ASCL1 could be playing a greater role in progenitor maintenance or differentiation in NB cell lines depending on the developmental position of the cell of origin of the original tumour.

For instance, a recent study showed that siRNA-mediated downregulation of ASCL1 in Kelly cells led to upregulation of NTRK1 and NPY, genes that are associated with differentiation (Wang et al., 2019). However, it seems likely that the absolute level of ASCL1 protein is critical to determine whether it supports proliferation or differentiation in a particular neuroblastoma cell line; siRNA knockdown led to a reduction of ASCL1 in Kelly cells, yet these cells have an endogenous level of ASCL1 that is considerably higher than IMR32 or SH-SY5Y cells, so significant ASCL1 protein remained even after knock-down (Wang et al., 2019). In our comparison between two neuroblastoma cell lines representatives of ALK-driven and MYCN-amplified disease, there is clearly heterogeneity of response to ASCL1 knockout despite similar initial expression levels. When comparing across cell lines, differences in gene expression between parental IMR32 and SH-SY5Y cells were greater than between each parental line and its ASCL1 KO derivative (Supplementary Figure S2A). Focussing on the changes in gene expression after ASCL1 deletion, 255 genes were downregulated in both cell lines, while a further 238 were downregulated in SH-SY5Y cells only, and 3148 were downregulated in IMR32 cells only (Figure 2C). Interestingly, genes enriched in only 1 cell line or another were also associated with neuronal processes such as synapse organisation and axonogenesis (Figure 2C), consistent with ASCL1 acting as a master regulator and playing a role in directing neuronal processes in general, yet regulating a different spectrum of targets depending on the differing neuronal identity of each cell line.

As PHOX2B, MYC/MYCN and other developmentally regulated transcription factors are key components of the CRC that supports the rapidly proliferating progenitor phenotype of neuroblastoma cells, and as ASCL1 KO cells divide more slowly than parental cells, we investigated whether ASCL1 deletion was downregulating expression of CRC components including MYC/MYCN that are known to support proliferation and oncogenicity (Durbin et al., 2018). We saw a generally insignificant or modest change in expression of transcripts of CRC components such as PHOX2B, GATA3 and HAND2 on ASCL1 KO (Figure 5A), which was not always consistent across both cell lines, indicating that ASCL1 is not absolutely required for maintaining high level expression of the core CRC network. This may be because one major role of ASCL1 is in maintaining chromatin accessibility particularly at genes associated with differentiation in the neuroblastic cells typifying neuroblastoma (Figure 3 and see below), rather than being required to maintain high level expression at genes controlled by open super-enhancers, which already have high concentrations of other transcriptional activators. However, despite limited changes in CRC gene transcription, closer analysis showed that cells lacking ASCL1 showed reduced overall chromatin binding of the critical CRC factors PHOX2B, GATA3 and MYC/MYCN (Figure 6B). Reduced binding of these factors is likely to contribute to the reduced proliferation of ASCL1 KO cells, particularly in the case of MYCN/MYC, whose role in driving proliferation in neuroblastoma is very well documented (Huang and Weiss 2013; Zimmerman et al., 2018; Otte et al., 2021).

To further test the hypothesis that ASCL1 regulates chromatin accessibility of these transcription factors, we utilised publicly available PHOX2B and GATA3 ChIP-seq datasets in parental BE2C and Kelly neuroblastoma cell lines (Durbin et al., 2018). Regulated genes were assigned by the closest transcription start site to either the ATAC-seq accessibility change or TF binding site. We have then overlapped these two gene sets based on the extent of change in the ATAC-seq using chromatin regions with no significant change in accessibility with ASCL1 knockout as a control for the comparison (ATAC-seq change: increased accessibility = log2FC > 0.5 & padj<0.05, decreased accessibility = log2FC < -0.5 & padj<0.05, n. s = padj>0.75). The results of this analysis show that a higher proportion of genes associated with significantly changed chromatin accessibility following ASCL1 knockdown are PHOX2B and GATA3 targets than genes for which there is no change in accessibility on ASCL1 KO (n.s group) (Supplementary Figure S7). This supports the hypothesis that ASCL1 knockout impacts the accessibility of GATA3 and PHOX2B binding sites.

Taken together, our data allows us to propose a model where endogenous ASCL1 plays two roles in neuroblastoma. Firstly, ASCL1 supports rapid proliferation probably at least in part by facilitating chromatin binding by the key proliferation drivers including MYC/MYCN (Wang et al., 2019; Ali et al., 2020). It appears to play this role distinct from an ability to drive expression of the MYC-driven CRC network of transcription factors (Wang et al., 2019; Alleboina et al., 2021). ASCL1 is a pioneer factor, able to open more inaccessible chromatin associated with differentiation genes, yet it may be less important for maintaining high level expression of genes controlled by super-enhancers that are already readily accessible and bound by multiple transcription factors (Boeva et al., 2017; van Groningen et al., 2017; Ponzoni et al., 2022), a super-enhancer network that is a hallmark of this trapped developmental intermediate stage seen in neuroblastoma tumours.

Deletion of ASCL1 reduces accessibility around loci associated with differentiated functions of neurons and inhibits expression of genes associated with a more mature neuronal identity including those associated with axonal outgrowth and synapse activity. In neuroblastoma cells a second function of endogenous ASCL1 is to keep chromatin around differentiation genes accessible. Thus, ASCL1 may act as a priming factor to facilitate activity of the cascade of transcriptional regulators that would direct progress of neuroblastic cells down their normal developmental trajectory. This function echoes ASCL1’s known role in fibroblast reprogramming, where it acts as a pioneer to open chromatin and facilitate the accessibility for additional transcription factors required to drive the process of reprogramming cells to a neuronal identity (Wapinski et al., 2013). In normal development rather than in the aberrant genetic circuits set up in cancer, PHOX2B works with other developmental transcription factors to drive noradrenergic neuron differentiation (Pattyn et al., 1999). We see that ASCL1 deletion reduces PHOX2B’s ability to bind chromatin, consistent with a model whereby ASCL1 is regulating access for this and other factors to a differentiation programme.

Thus ASCL1’s endogenous role in neuroblastoma cells echoes its normal developmental role sitting at the critical nexus balancing proliferation and differentiation in neuroblasts. While playing a redundant role in supporting the oncogenic CRC gene expression (Figures 5, 6), ASCL1 instead appears to play a key role in priming neuroblastoma cells for differentiation (Figure 2). We have previously seen that hyperactivation of ASCL1 can result in precocious cell cycle exit and differentiation (Ali et al., 2020; Woods et al., 2022). A better understanding of ASCL1’s function in these cells may lead to therapies that activate the latent ability of neuroblastoma cells to undergo differentiation for patient benefit.

### Permission to reuse and copyright

Open Access This article is licensed under a Creative Commons Attribution 4.0 International License, which permits use, sharing, adaptation, distribution and reproduction in any medium or format, as long as you give appropriate credit to the original author(s) and the source, provide a link to the Creative Commons licence, and indicate if changes were made. The images or other third party material in this article are included in the article’s Creative Commons licence, unless indicated otherwise in a credit line to the material. If material is not included in the article’s Creative Commons licence and your intended use is not permitted by statutory regulation or exceeds the permitted use, you will need to obtain permission directly from the copyright holder. To view a copy of this licence, visit http://creativecommons.org/licenses/by/4.0/.

## Data Availability

All the ATAC-seq and RNA-seq data used for this publication have been deposited on GEO under the SuperSeries GSE202635. The RNA-seq data SubSeries has been assigned the identifier GSE202634 and the ATAC-seq data SubSeries has been assigned the identifier GSE202629.

## Abbreviations

ADRN, Adrenergic; ALK, Anaplastic Lymphoma Kinase; ASCL1, Achaete-Scute Homolog 1; ATAC, Assay for Transposase Accessible Chromatin; CNS, Central Nervous System; CRC, Core Regulatory Circuit; GATA3, GATA binding protein 3; GO, Gene Ontology; GREAT, Genomic Regions Enrichment of Annotations Tool; GSEA, Gene Set Enrichment Analysis; KO, Knockout; MES, Mesenchymal; PCA, Principal Component Analysis; PHOX2B, Paired-like homeobox 2b; PNS, Peripheral Nervous System; TSS, Transcriptional Start Site.
