# Supplementary material for: The proneural transcription factor ASCL1 regulates cell proliferation and primes for differentiation in neuroblastoma
Source: Front Cell Dev Biol. 2022 Oct 3;10:942579. doi: 10.3389/fcell.2022.942579 (PMC9574099; doi:10.3389/fcell.2022.942579)
Supplement: Supplementary file 1 [file DataSheet1.pdf]

Supplementary Figure 1

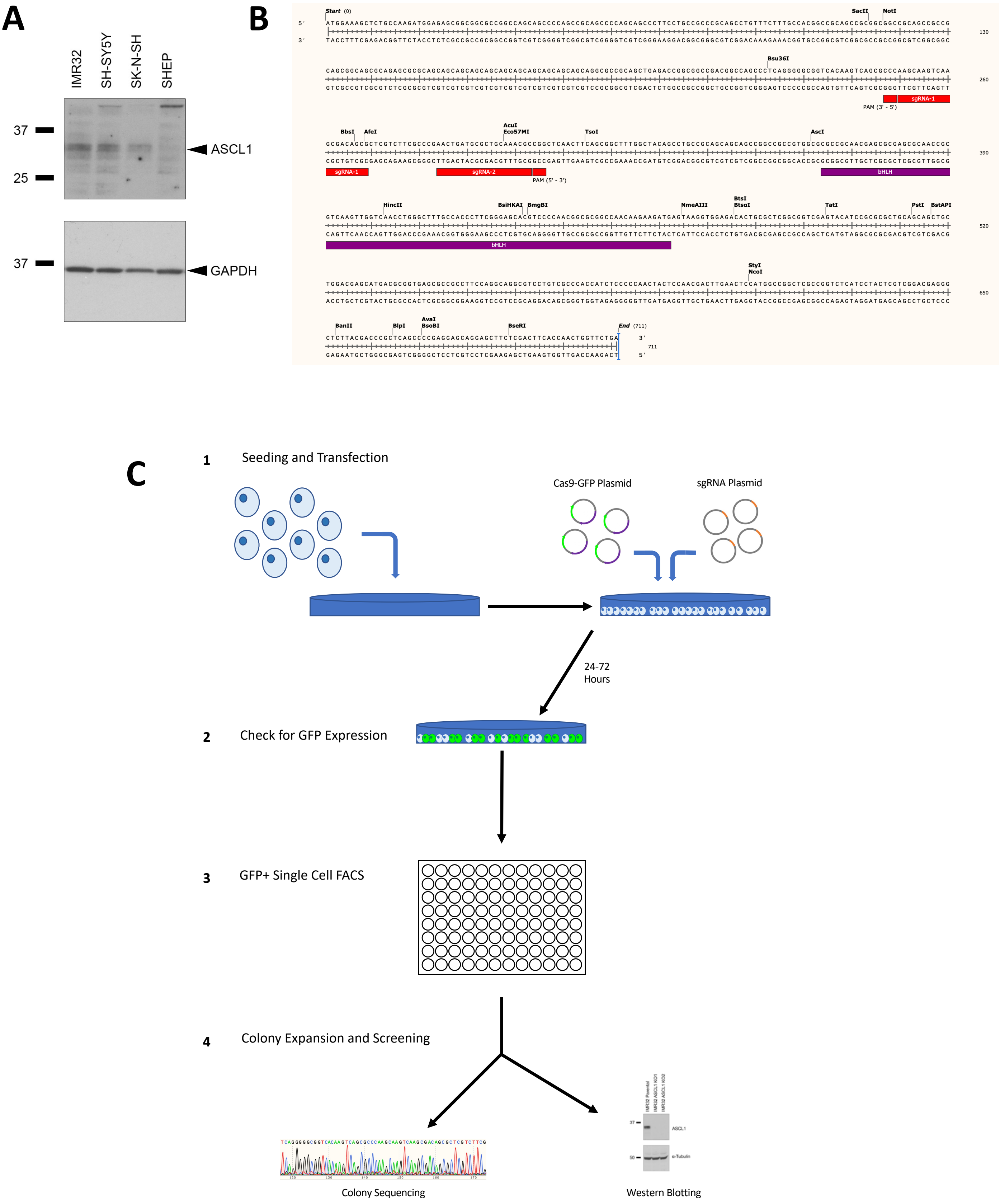

Supplementary Figure 1 (Continued)

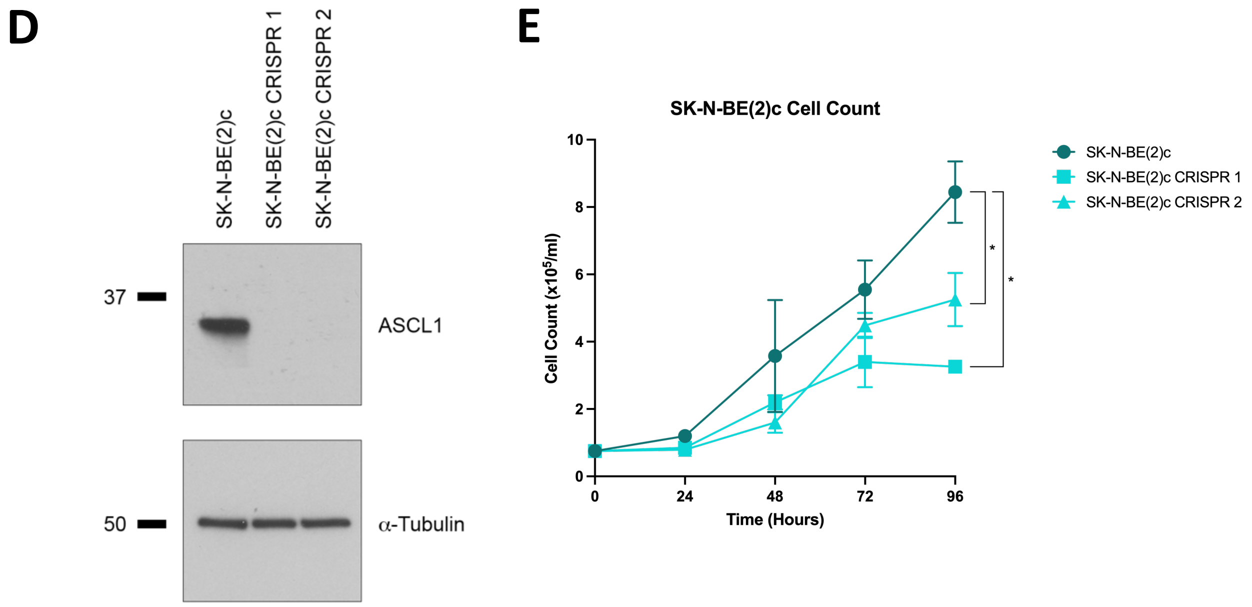

Supplementary Figure 1: ASCL1 Knockout Process. (A) Western blot to show levels of ASCL1 protein in IMR32 and SH-SY5Y cell lines. (B) The sgRNA targeted a location of the gene upstream of the bHLH. (C) Schematic of ASCL1 knockout (KO), beginning with seeding of cells and transfection of the CRISPR plasmids. Transfected cells were left for between 24 and 72 hours and checked for GFP expression. GFP positive cells were then single cell sorted into 96 well plates, colonies were expanded for 4-6 weeks before colony screening by sequencing and western blot. (D) Western blotting to show the ASCL1 levels in SK-N-BE(2)c parental lines and in lines following ASCL1 KO; α-Tubulin was used as the loading control. (E) Cell count data of SK-N-BE(2)-C parental and ASCL1 KO cells over a 96-hour period. n=3, graphs show mean value and +/- SEM (unpaired two-tailed t-test * = $p < 0.05$).

Supplementary Figure 2

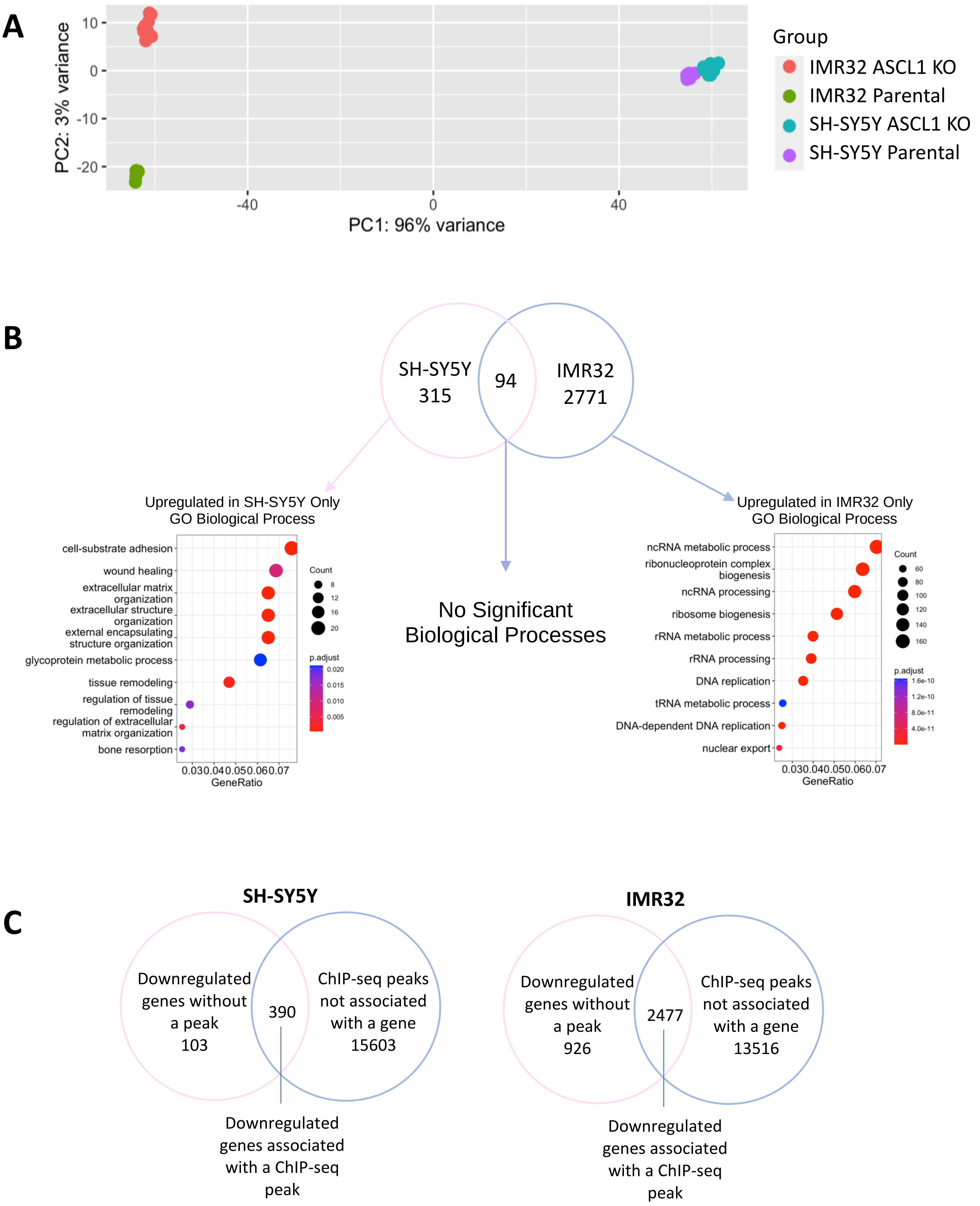

Supplementary Figure 2 (continued)

D

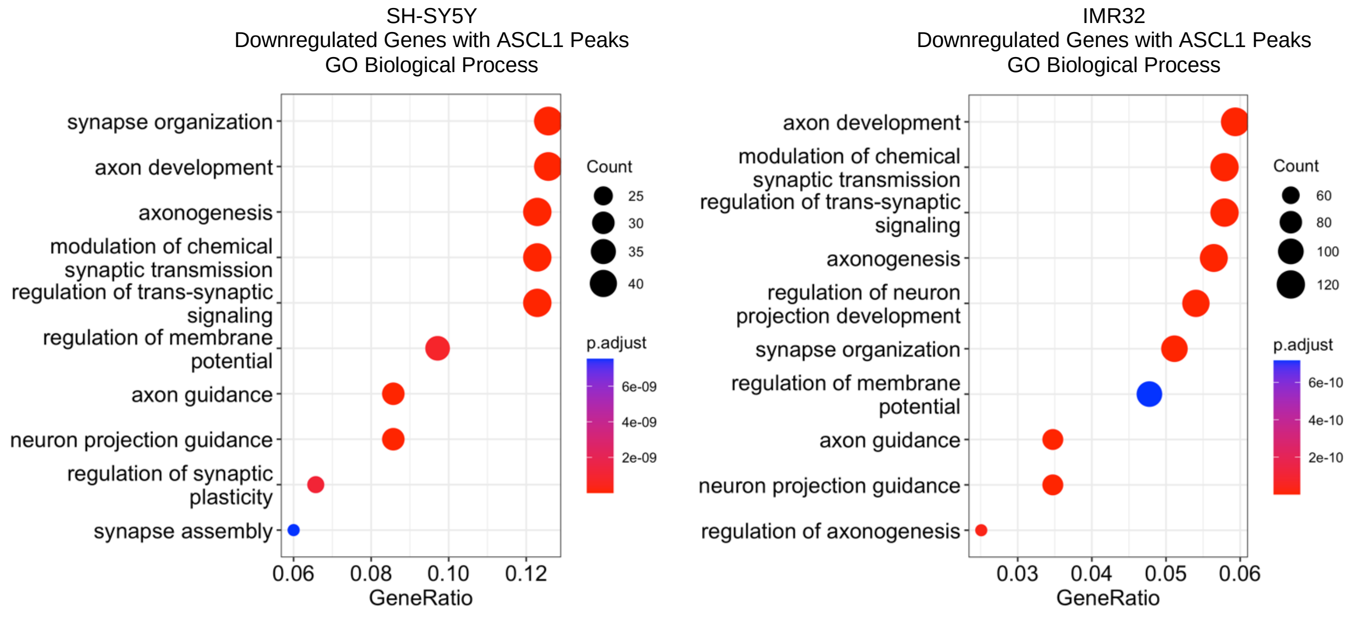

Supplementary Figure 2: (A) PCA Analysis of RNA-seq data from IMR32 and SH-SY5Y parental and KO cells. (B) Venn diagram to show the genes upregulated in SH-SY5Y ASCL1 KO lines, IMR32 ASCL1 KO lines and the genes upregulated in both. GSEA analysis was completed on each gene set. The top 10 GO terms associated with biological process are shown. (C) Venn diagram to show the genes downregulated in SH-SY5Y ASCL1 KO lines (left) and IMR32 ASCL1 KO lines (right) that are associated with an ASCL1 overexpression ChIP-seq peak. (D) GSEA analysis performed on the downregulated genes in ASCL1 KO lines that also are associated with an ASCL1 ChIP-seq peak. The top 10 GO terms are shown.

Supplementary Figure 3

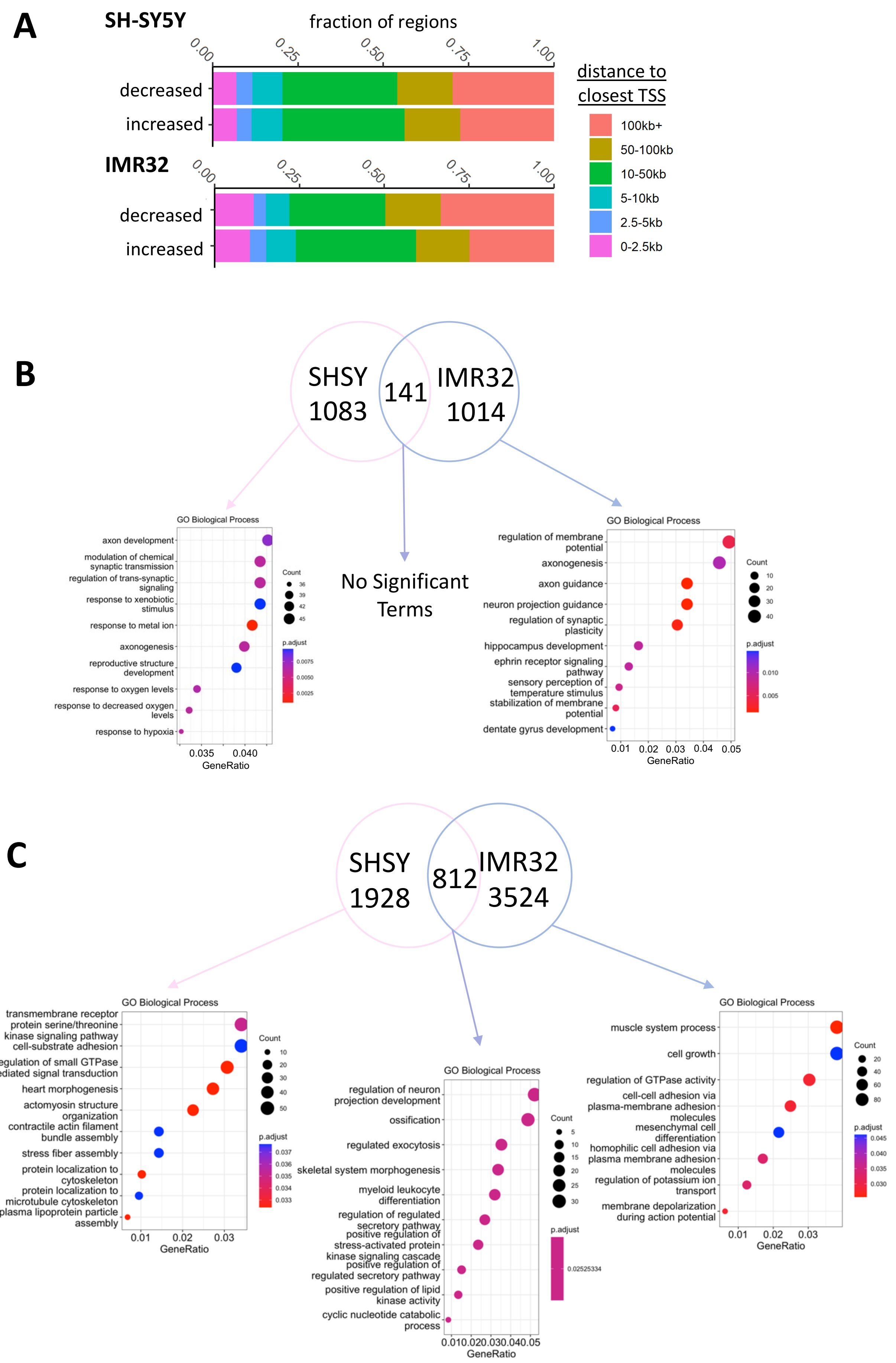

Supplementary Figure 3: Analysis of regions identified as differentially accessible following ASCL1 KO. (A) Proximity of the closest transcription start sites to the regions with significantly altered accessibility following ASCL1 knockdown. (B) Genes within 50kb of an ATAC region with reduced accessibility were identified. Genes were sectioned depending on whether they were less accessible in only SH-SY5Y, only IMR32 or less accessible across all KO lines. (C) Genes within 50kb of an ATAC region with increased accessibility were identified. Genes were sectioned depending on whether they were more accessible in only SH-SY5Y, only IMR32 or more accessible across all KO lines. Gene Ontology analysis was completed on each gene set. The top 10 GO terms associated with biological process are shown.

Supplementary Figure 4

A

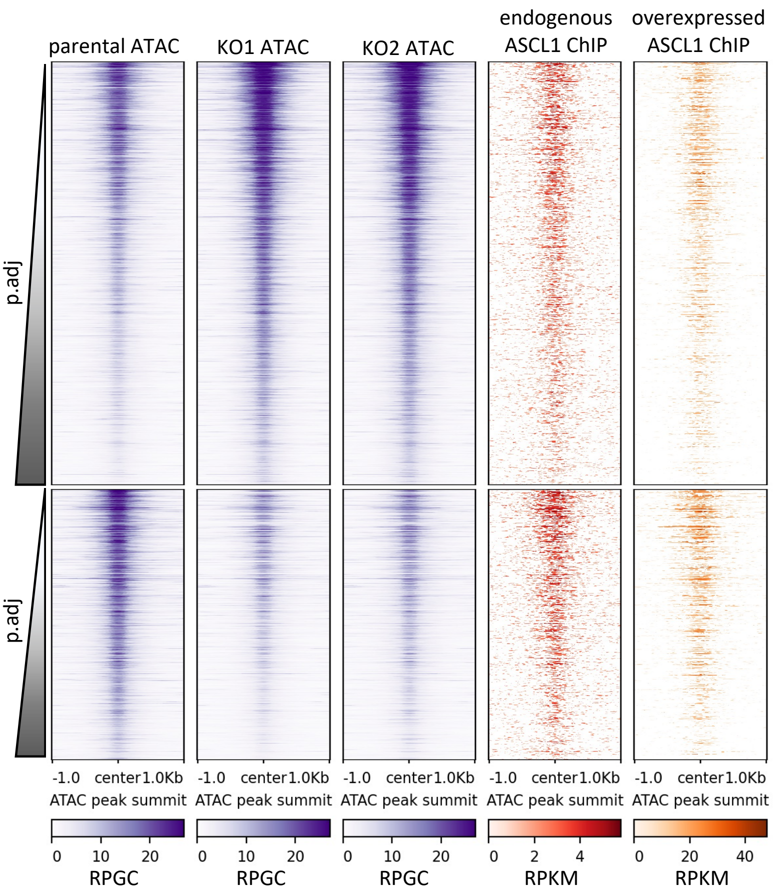

B

ASCL1 binding site & ↓ accessibility

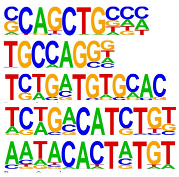

de novo motifs	p-value	% of targets	% of background	similar binding motifs
	1x10^{58}	91.28	38.90	bHLH proteins, incl. ASCL1
	1x10^{16}	50.46	23.83	ZBTB29
	1x10^{14}	6.42	0.26	ZSCAN4
	1x10^{14}	9.63	0.96	HAND2, TCF4
	1x10^{13}	5.96	0.28	PGR, NR3C1

C

no binding site & ↓ accessibility

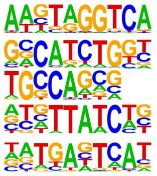

de novo motifs	p-value	% of targets	% of background	similar binding motifs
	1x10^{166}	17.52	2.69	RORG, RORC, RORA
	1x10^{69}	48.35	29.32	bHLH proteins, incl. TCF4, HAND2, NEUROD1
	1x10^{48}	52.46	36.23	ZBTB29
	1x10^{46}	27.85	15.14	TRPS1, GATA proteins
	1x10^{45}	14.89	5.98	bZIP proteins incl. FOS, JUNB

D

ASCL1 binding site & ↑ accessibility

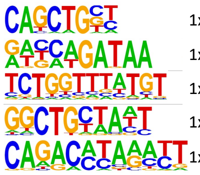

de novo motifs	p-value	% of targets	% of background	similar binding motifs
	1x10^{58}	84.21	27.84	bHLH proteins, incl. ASCL1
	1x10^{21}	27.37	5.62	TRPS1, GATA proteins
	1x10^{16}	26.84	6.82	HAND2
	1x10^{12}	21.05	5.54	DCE, ZNF341
	1x10^{12}	28.42	9.92	SMAD proteins

E

no binding site & ↑ accessibility

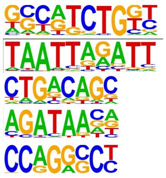

de novo motifs	p-value	% of targets	% of background	similar binding motifs
	1x10^{177}	62.90	38.10	bHLH proteins, incl. HAND2, NEUROD1, TCF4
	1x10^{146}	38.37	18.84	homeobox proteins, incl. PHOX2A, PROP1, PHOX2B
	1x10^{91}	63.61	45.77	MEIS1, MEIS2, TGIF1
	1x10^{66}	32.80	19.83	TRPS1, GATA proteins
	1x10^{64}	56.55	41.60	ZFX, ZNF711

Supplementary Figure 4: Differentially accessible regions following ASCL1 KO are associated with an ASCL1 ChIP-Seq Peak. Signal intensity -/+1kb around peak summits of all regions with altered accessibility in SH-SY5Y ASCL1 KO cell lines (p.adj<0.05). The heatmaps are ordered by the extent of the significance of the change in accessibility using the FDR value from DiffBind (most significant to least significant). All heatmaps are centred around the summit of the region with changed accessibility. Purple shows the ATAC-seq data in parental and ASCL1 KO lines (RPGC normalised), red shows the signal from endogenous ASCL1 ChIP-seq (uninduced clone) and orange shows the signal for overexpressed WT ASCL1 (ChIP-seq data RPKM normalised from Ali et al. 2020). (B-E) Five most significant de novo motifs determined using HOMER for motif analysis. Similar binding motifs denote proteins with known motifs that align with high similarity to the de novo motifs. Motif analysis is shown for (B) regions with decreased accessibility and a significant endogenous ASCL1 binding site (C) regions with decreased accessibility but no significant endogenous ASCL1 binding (D) regions with increased accessibility and a significant endogenous ASCL1 binding site (E) ATAC regions with increased accessibility but no significant endogenous ASCL1 binding.

Supplementary Figure 5

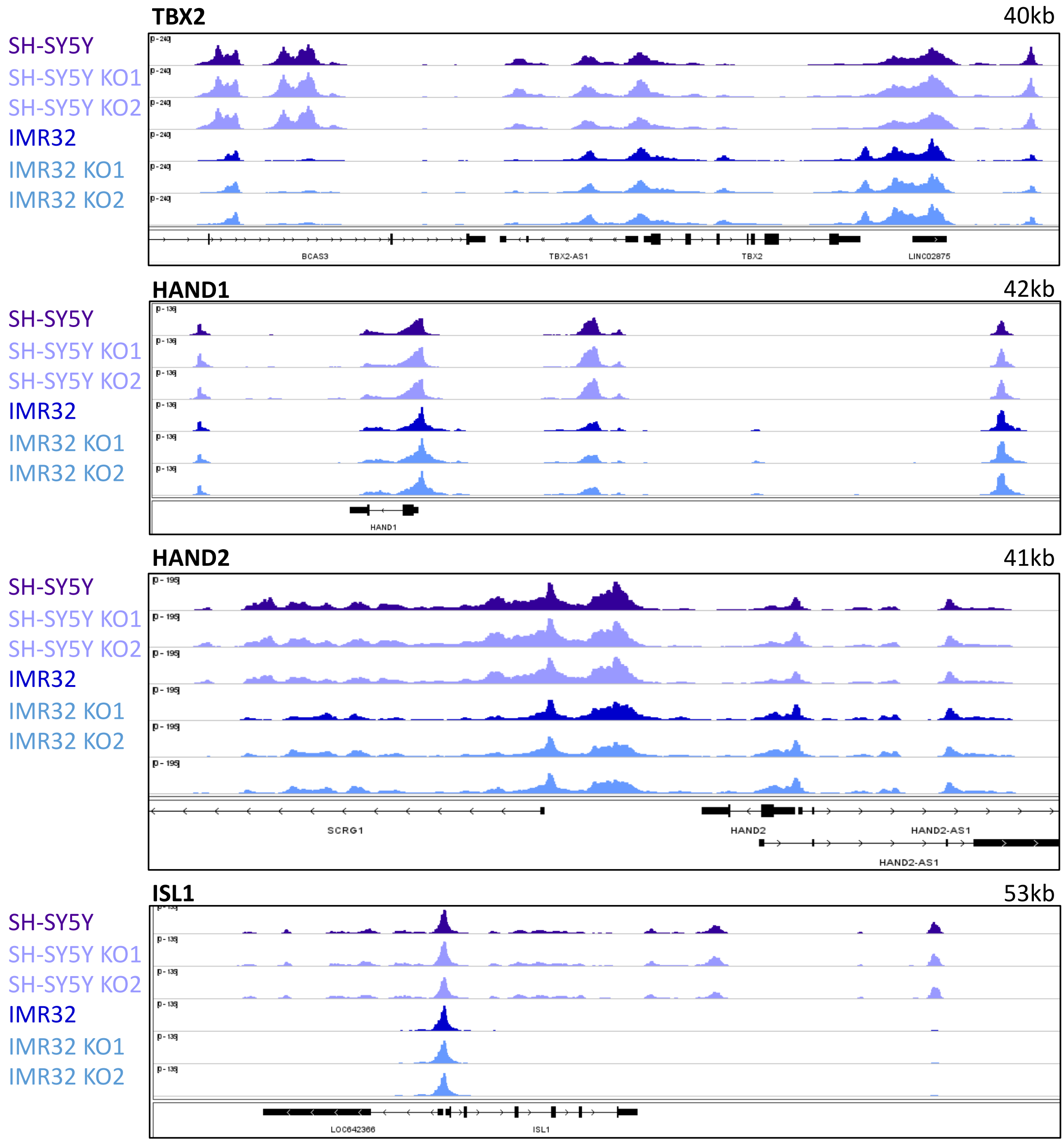

Supplementary Figure 5: Chromatin accessibility around key ADRN CRC TFs is unchanged. ATAC-Seq tracks showing regions around the key TFs TBX2, HAND1, HAND2 and ISL1. ATAC-Seq was completed on 4 biological replicates.

Supplementary Figure 6

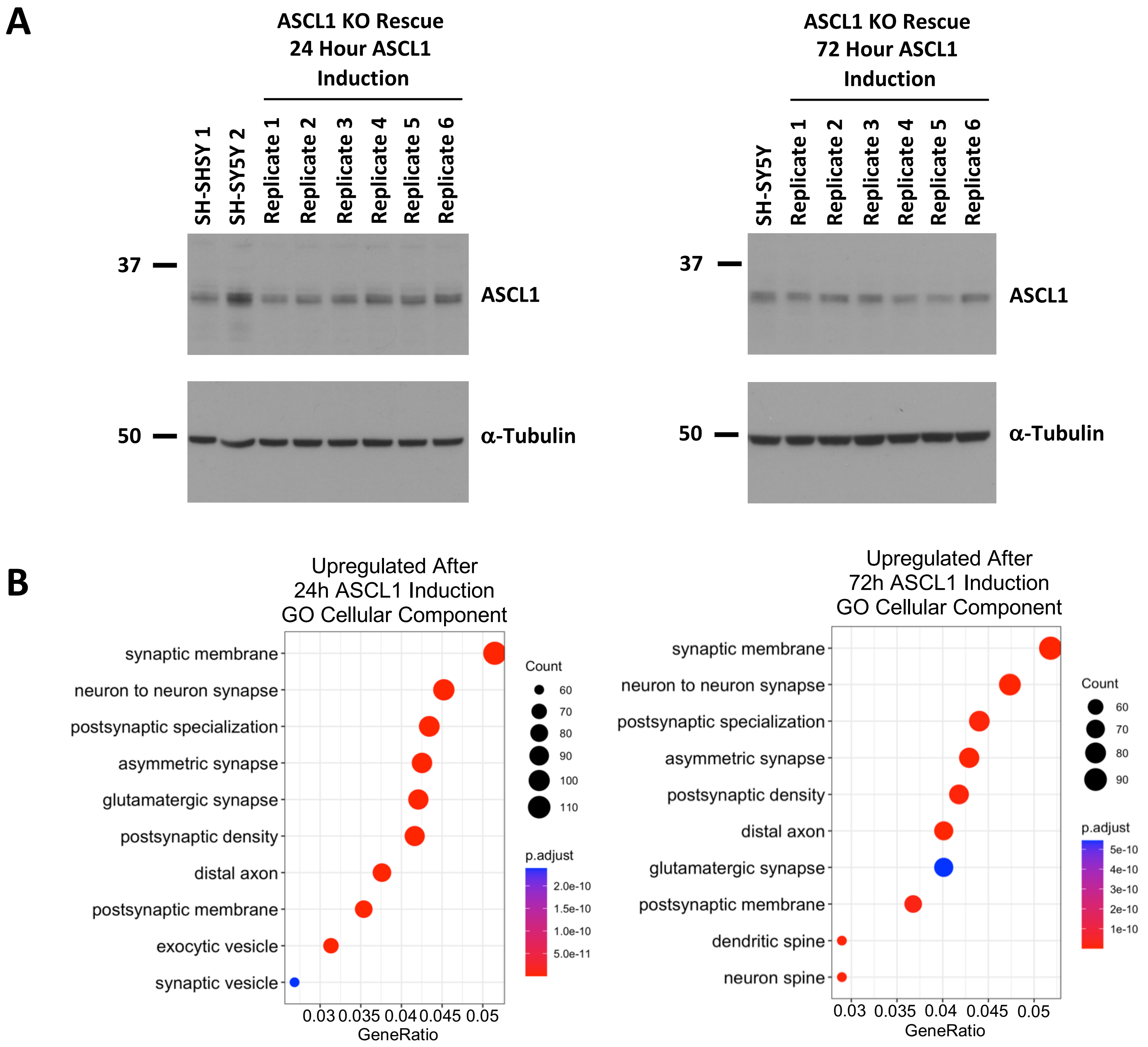

Supplementary Figure 6: (A) Western blots to show the level of ASCL1 in the rescue lines following induction with 5ng/ml Doxycycline for 24 or 72 hours. (B) gsea analysis was performed on the upregulated genes identified as differentially expressed following 24 hours (left) and 72 hours (right) ASCL1 induction. The top 10 GO terms associated with cellular component are shown.

Supplementary Figure 7

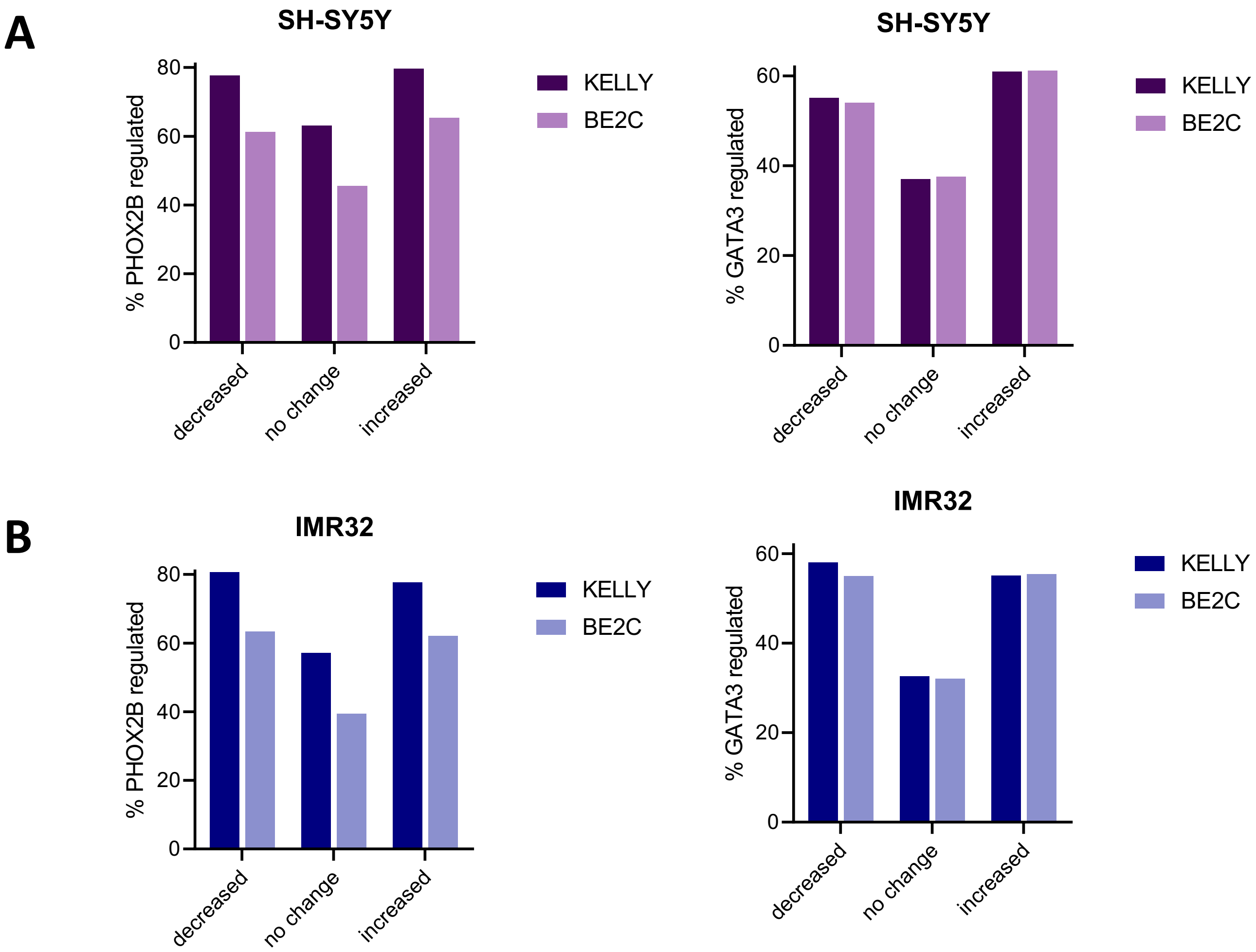

Supplementary Figure 7: Differentially accessible regions after ASCL1 knockout are more associated with PHOX2B and GATA3 binding sites. Graphs show the percentage overlap of genes that have an associated PHOX2B or GATA3 binding site (data from BE2C and Kelly cells Durbin et al. 2018) with genes that have an associated ATAC regions separated by the impact of ASCL1 KO on accessibility (ATAC-seq change : increased accessibility = log2FC>0.5 & p.adj<0.05, decreased accessibility = log2FC<-0.5 & p.adj<0.05, no significant change = p.adj>0.75). This is shown for the ATAC-seq data from (A) SH-SY5Y cells (B) IMR32 cells.
